# Genome-wide characterization of post-transcriptional processes related to wood formation in *Dalbergia odorifera*

**DOI:** 10.1186/s12864-024-10300-7

**Published:** 2024-04-16

**Authors:** Nanbo Jiao, Jieru Xu, Yue Wang, Dunxi Li, Feifei Chen, Yu Chen, Jinhui Chen

**Affiliations:** 1 School of Breeding and Multiplication (Sanya Institute of Breeding and Multiplication), School of Tropical Agriculture and Forestry, Hainan University, Sanya, 572019 China; 2 Hainan Academy of Forestry (Hainan Academy of Mangrove), Haikou, 571100 China

**Keywords:** *Dalbergia Odorifera*, Alternative polyadenylation, Alternative splicing, lncRNAs, Iso-Seq, Wood formation

## Abstract

**Background:**

Alternative polyadenylation (APA), alternative splicing (AS), and long non-coding RNAs (lncRNAs) play regulatory roles in post-transcriptional processes in plants. However, little is known about their involvement in xylem development in *Dalbergia odorifera*, a valuable rosewood species with medicinal and commercial significance. We addressed this by conducting Isoform Sequencing (Iso-Seq) using PacBio’s SMRT technology and combined it with RNA-seq analysis (RNA sequencing on Illumina platform) after collecting xylem samples from the transition zone and the sapwood of *D. odorifera*.

**Results:**

We identified 14,938 full-length transcripts, including 9,830 novel isoforms, which has updated the *D. odorifera* genome annotation. Our analysis has revealed that 4,164 genes undergo APA, whereas 3,084 genes encounter AS. We have also annotated 118 lncRNAs. Furthermore, RNA-seq analysis identified 170 differential alternative splicing (DAS) events, 344 genes with differential APA site usage (DE-APA), and 6 differentially expressed lncRNAs in the transition zone when compared to the sapwood. AS, APA, and lncRNAs are differentially regulated during xylem development. Differentially expressed APA genes were enriched for terpenoid and flavonoid metabolism, indicating their role in the heartwood formation. Additionally, DE-APA genes were associated with cell wall biosynthesis and terpenoid metabolism, implying an APA’s role in wood formation. A DAS gene (involved in chalcone accumulation) with a significantly greater inclusion of the last exon in the transition zone than in the sapwood was identified. We also found that differentially expressed lncRNAs targeted the genes related to terpene synthesis.

**Conclusions:**

This study enhances our understanding of the molecular regulatory mechanisms underlying wood formation in *D. odorifera*, and provides valuable genetic resources and insights for its molecular-assisted breeding.

**Supplementary Information:**

The online version contains supplementary material available at 10.1186/s12864-024-10300-7.

## Background

*Dalbergia odorifera* T. Chen, also known as Hainan Huanghuali, is a precious tree species that belongs to the family Leguminosae, and is mainly found in Hainan, China. It possesses significant economic value due to its hard, moisture- and corrosion-resistant stem core, and a display of fine and beautiful patterns. It has an obvious reddish-brown or dark red color; its wood is shiny and has a special aromatic smell. Moreover, the heartwood of *D. odorifera* also serves as a vital raw medicinal material, as it contains terpenoids and flavonoids that contribute to its pharmacological significance [1]. However, the slow natural formation of the heartwood of *D. odorifera* [1] hinders the utilization of such a valuable resource. Although some methods for the artificial induction of heartwood formation have been reported, the mechanism underlying its formation remains unclear [1]. Previous studies have focused on phytochemistry of the heartwood formation process in *D. odorifera* [1, 2], while the molecular regulatory mechanism governing wood formation in this species has largely remained unexplored.

Polyadenylation, an important step in the precursor mRNA processing, serves as a crucial post-transcriptional regulatory mechanism. It is primarily mediated by alternative polyadenylation (APA), which produces multiple isoforms by utilizing different poly(A) sites. This alters the coding ability of an mRNA, and/or influences the stability of proteins or RNAs, and thus plays a critical role in the modulation of gene expression [3]. For example, a recent study on *Spartina alterniflora* revealed that APA generates alternative 3’ UTRs of the HIGH-AFFINITY K + TRANSPORTER 1 (*SaHKT1*). This increases the stability of its RNA and promotes protein synthesis, thus regulating the plant’s response to environmental stresses [4]. Multiple studies have highlighted a significant role of APA in plant development and stress response [3–5]. However, the expression and implications of APA in wood formation in trees is little known.

Alternative splicing (AS) of a precursor mRNA is also an important process that regulates the expression of genes at the post-transcriptional level. It increases the diversity of a transcriptome and a proteome, and plays a significant role in regulating a plant’s phenotype [6, 7]. For example, there is an early study suggested a causal link between junction sequence, alternative splicing, and isochorismate synthase function [8]. In another previous study, AS events of *SND1* was known as regulating secondary xylem development, which was corresponded with wood formation [9]. A recent study in a tea plant (*Camellia sinensis*) showed that, *CsJAZ1* produces a full-length transcript (CsJAZ1-1) and two splice variants (CsJAZ1-2 and -3) that lack 3’ coding sequences, where CsJAZ1-3 also lacks the coding region for the Jas domain. These alternative splicing variants of *CsJAZ1* harmoniously regulate the biosynthesis of flavan-3-ol in a tea plant [10]. Although AS is an important post-transcriptional regulatory mechanism, its characterization in *D. odorifera* is still lacking.

Long non-coding RNAs (lncRNAs) are another important regulator of plant growth and development [11–13]. For example, lncRNA *MISSEN*, a parent-of-origin lncRNA in rice, regulates the function of tubulin by hijacking a helicase family protein, HeFP. This negatively regulates endosperm development, resulting in obvious depressions and bulges in the seeds [14]. However, roles of lncRNAs in the growth and development of *D. odorifera*, including that of wood formation is still unknown.

Wood can be divided into three parts: sapwood, transition zone and heartwood. The sapwood is an outer zone in the cross-section of stem of a mature tree. It is characterized by its light color and a layer of younger growth rings that contain actively functioning cells. Whereas, the heartwood is darker, inner zone, which is characterized by a section of older growth rings that contain non-active cells [15]. The heartwood tissues are formed through the progressive transformation of the innermost growth rings of the sapwood, which initially occurs in the intermediary region, commonly referred to as the transition zone [15]. Typically, the transition zone is located immediately next to the heartwood and spans a width of one to two growth rings [16]. The sapwood and transition zone contains living parenchyma cells, but heartwood is formed once the cells die [15, 17]. Hence, the xylem tissue from the transition zone holds significant value as a material for investigating wood formation.

The contents of the transition zone have been extracted and characterized for investigating the processes involved in heartwood formation [18, 19]. In *D. odorifera*, flavonoids and terpenoids constitute the primary secondary metabolites within the heartwood [1, 2, 18]. Cellulose, hemicellulose, lignin and pectin are the major components of a wood while the cell wall is essential to the development of a wood due to its richness in the major components of a wood [17]. Thus, the synthesis of cell wall contributes to wood formation while programmed cell death contributes to heartwood formation; further, the synthesis of flavonoids and terpenoids contributes to the characteristics of heartwood of *D. odorifera*. However, the molecular mechanisms underlying these processes have remained poorly understood due to a poor understanding of the genes that could be involved in the process.

Although the genome of *D. odorifera* had been published in 2020 [20], the research on its transcriptome is still lacking. In this study, we employed the Iso-Seq method to explore the intricate transcriptome of the *D. odorifera*. Since the development of technology, the Iso-Seq method of third-generation sequencing (from Pacific Biosciences or PacBio) is offering great advantages for the whole transcriptome profiling. It is enabling the generation of full-length sequences of isoforms without the need of assembly (of short reads), thereby providing comprehensive insights into transcript diversity and isoform representation [21]. It increases the chances for discovering new genes and detecting AS events, APA, as well as lncRNAs. The combined application of second- and third-generation sequencing technologies has emerged as a powerful tool in transcriptomics research, successfully revealing transcriptome and gene structure information in multiple plant species [22–25].

Here, we have comprehensively characterized the sapwood and transition zones of *D. odorifera* at molecular level, where novel transcripts and corresponding genes were identified. This has also helped us to update the existing reference transcript dataset of this species. Furthermore, post-transcriptional processing of pre-mRNAs, including APA, AS, and the role of lncRNAs, was systematically analyzed. To uncover the divergence of AS variants and APA events among different zones of xylem, transcriptional and post-transcriptional expression patterns of sapwood and transition zone were analyzed using Illumina sequencing. The results revealed that several genes related to wood formation are regulated by APA, AS or lncRNA in *D. odorifera*. This offers a fresh perspective on the understanding of the molecular regulatory events involved in wood formation in this species.

## Results

### An overview of Iso-Seq analysis

Here, we used the Iso-Seq method to conduct transcriptome analysis of *D. odorifera*. RNA was extracted from the sapwood and the transition zone of a tree, which has formed the heartwood, and libraries were made. From these two libraries, Iso-Seq produced a total of 307,642 and 222,816 polymerase reads, respectively, which formed 9,958,487 and 8,372,271 subreads, with an average read length of 1,942 and 2,780 nucleotides respectively, and N50 of 2,115 and 3,160, respectively (Table 1). To obtain a more accurate sequence information, 255,411 and 201,632 circular consensus sequences (CCSs) respectively, were then generated from reads that passed at least 2 times through the insert (Table 1). Of these CCSs, a total of 183,972 and 221,632 sequences respectively, were identified as full-length non-chimeric reads (FLNC), which exhibited low artificial concatemers, and contained 5’- and 3’- primers as well as poly(A) tail (Table 1). The FLNC sequences belonging to the same transcript were clustered by using iterative clustering and error correction (ICE) algorithm (PacBio SMRT Link v6.0) to generate consensus sequences. These were polished with the help of the ‘arrow’ software (PacBio SMRT Link v6.0). This generated 23,386 and 20,758 polished consensus sequences, respectively for subsequent analysis. To enhance the accuracy of the FLNC reads, we further corrected all the polished consensus isoforms with Illumina short reads with the help of the LoRDEC software (PacBio SMRT Link v6.0). This produced a total of 23,386 and 20,758 high quality corrected consensus sequences, respectively, with a mean length of 2,155 and 3,065 nt (nucleotides), respectively, and a N50 of 2,352 and 3,397, respectively (Table 1).

**Table 1:** Summary of reads from Isoform Sequencing

	Number			Mean length			N50	
	DoSW	DoTZ		DoSW	DoTZ		DoSW	DoTZ
Polymerase Reads	307,642	222,816		65,278	107,245		146,239	169,418
Subreads	9,958,487	8,372,271		1,942	2,780		2,115	3,160
Circular consensus sequences	255,411	201,632		2,216	3,251		2,396	3,602
Full-length non-chimeric reads	221,632	183,972		2,089	3,138		2,286	3,513
Polished consensus	23,386	20,758		2,155	3,065		2,352	3,397
Corrected Consensus	23,386	20,758		2,155	3,065		2,352	3,397

DoSW: sapwood; DoTZ: transition zone; N50: the length of the longest contig such that all contigs of at least that length compose at least 50% of the bases of the assembly.

**Fig. 1:**
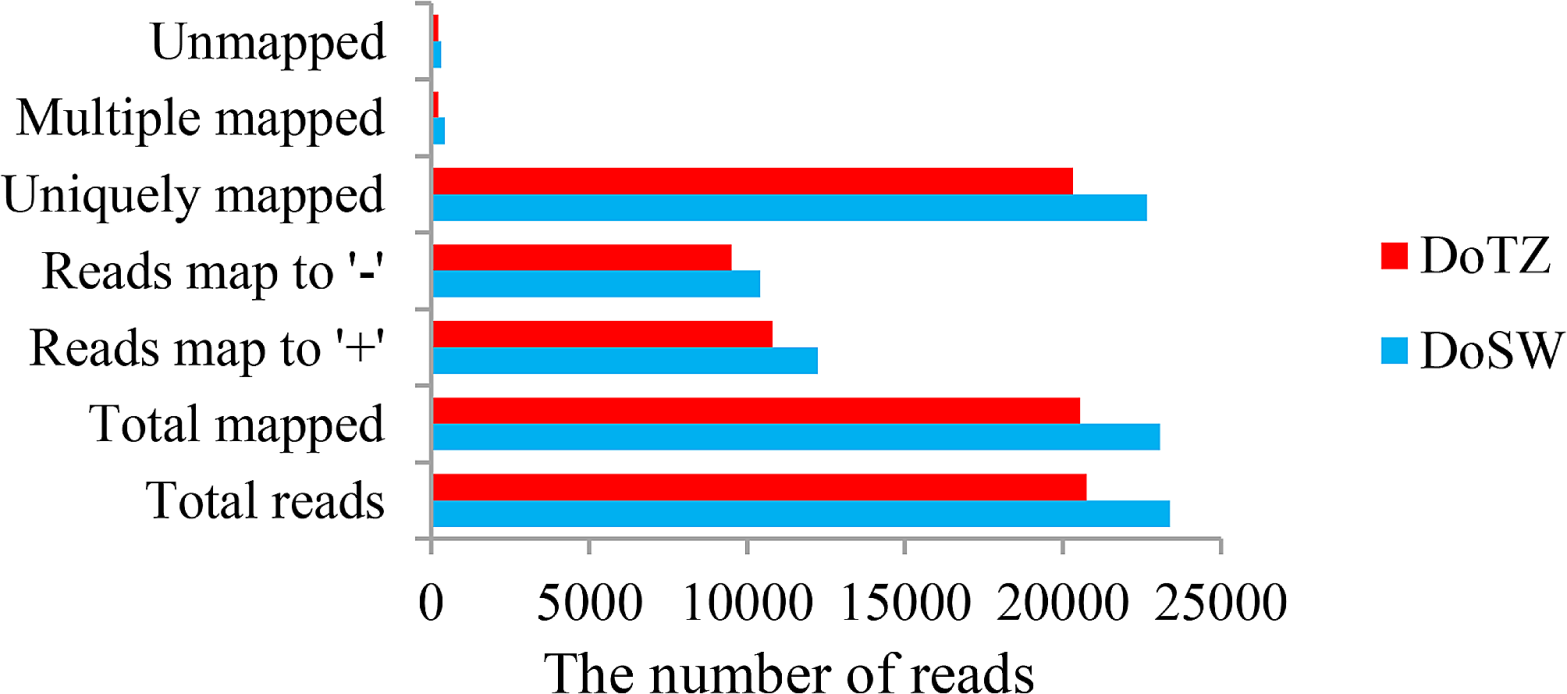
Statistics of GMAP mapping in DoSW (sapwood) and DoTZ (transition zone) tissues

The corrected consensus reads were then aligned to the genome sequence of *D. odorifera* [20] by using GMAP software [26]. A total of 23,074 (98.67%) and 20,542 (98.96%) reads were mapped to the reference genome in DoSW (sapwood) and DoTZ (transition zone) samples, respectively. Based on the mapping results, these reads were categorized into the following five groups: unmapped, multiple mapped, uniquely mapped, mapped to ‘+’ and mapped to ‘-’ strands. (Fig. 1).

### Identification of novel genes and transcripts

**Fig. 2:**
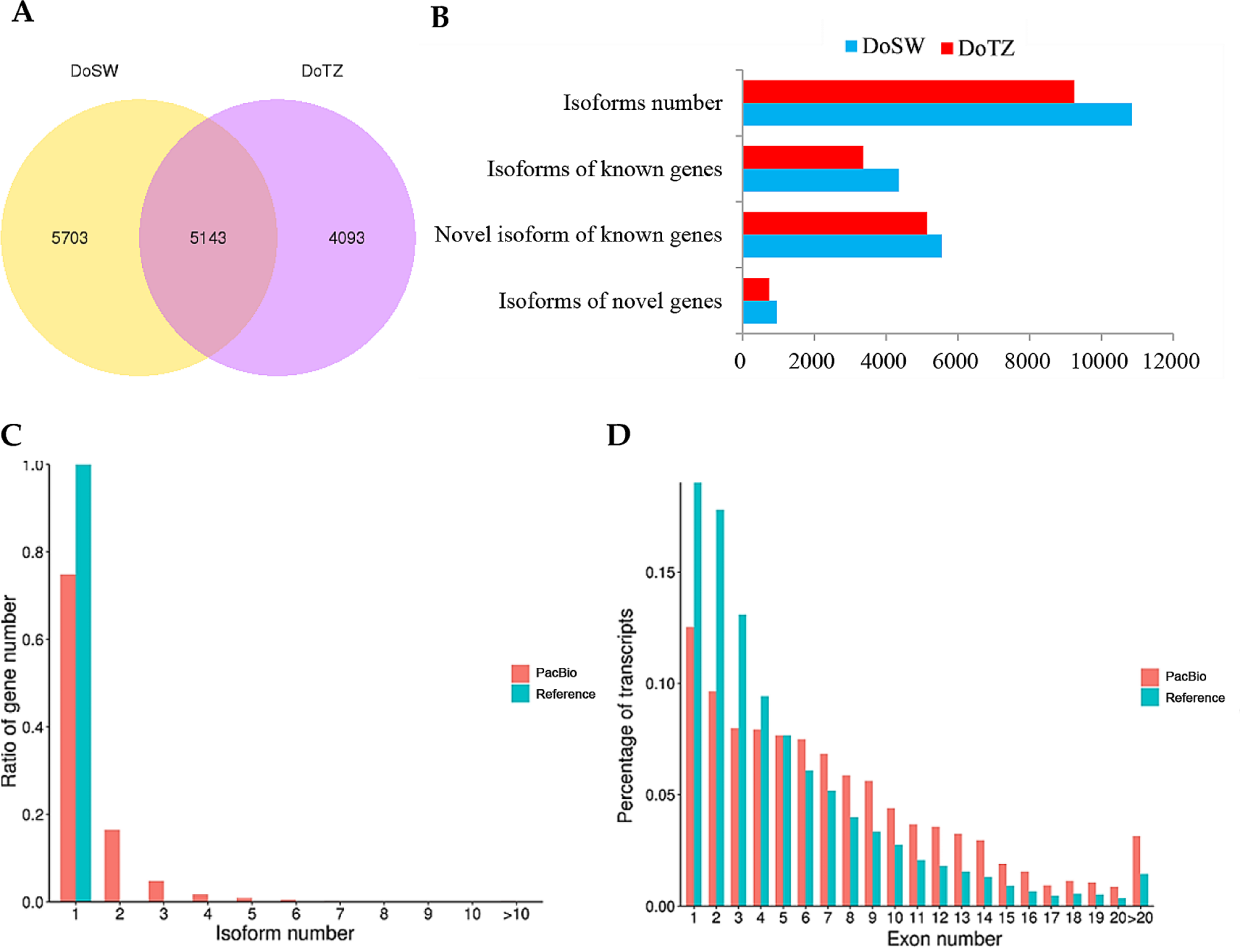
Iso-Seq identified large numbers of new transcripts in *D. odorifera*. (**A**) The Venn diagram shows the number of transcripts identified in DoSW (sapwood) and DoTZ (transition zone) samples, as well as those shared between the two. (**B**) A large number of novel transcripts were found in DoSW and DoTZ. (**C**) Distribution of the percentage of genes with different isoform numbers for reference and Iso-Seq data is shown. (**D**) The distribution of transcripts with varying numbers of exons for both reference dataset and Iso-Seq data is also presented

Here, we used the transcriptome analysis pipeline for Isoform Sequencing (TAPIS) [27] to identify unannotated genes. Reads that aligned to distinct exons within the annotated genes were categorized as belonging to novel isoforms. On the other hand, reads that aligned to the unannotated regions of the reference genome were regarded as belonging to transcripts of novel genes. From the PacBio Iso-Seq data, we detected 10,845 and 9,235 transcripts from DoSW and DoTZ, respectively (Fig. 2A). A total of 9,830 novel isoforms (6,495 in DoSW and 5,878 in DoTZ) were found, while 1,362 (946 in DoSW and 734 in DoTZ) reads were assigned to novel genes that had no overlap with any annotated region (Fig. 2B). Apparently, more isoforms were detected in DoSW than in DoTZ. A large number of sample-specific transcripts, which were richer in DoSW (5,703) than DoTZ (4,093), were found (Fig. 2A). Also, we found a larger number of genes with complex transcript constitutions, as well as transcripts with more complex exon constitutions, as compared with those in the reference genome (Fig. 2C and D). All of our newly discovered isoforms as well as the existing isoforms in the reference gene set [24] were used for subsequent analysis.

### Alternative polyadenylation analysis

**Fig. 3:**
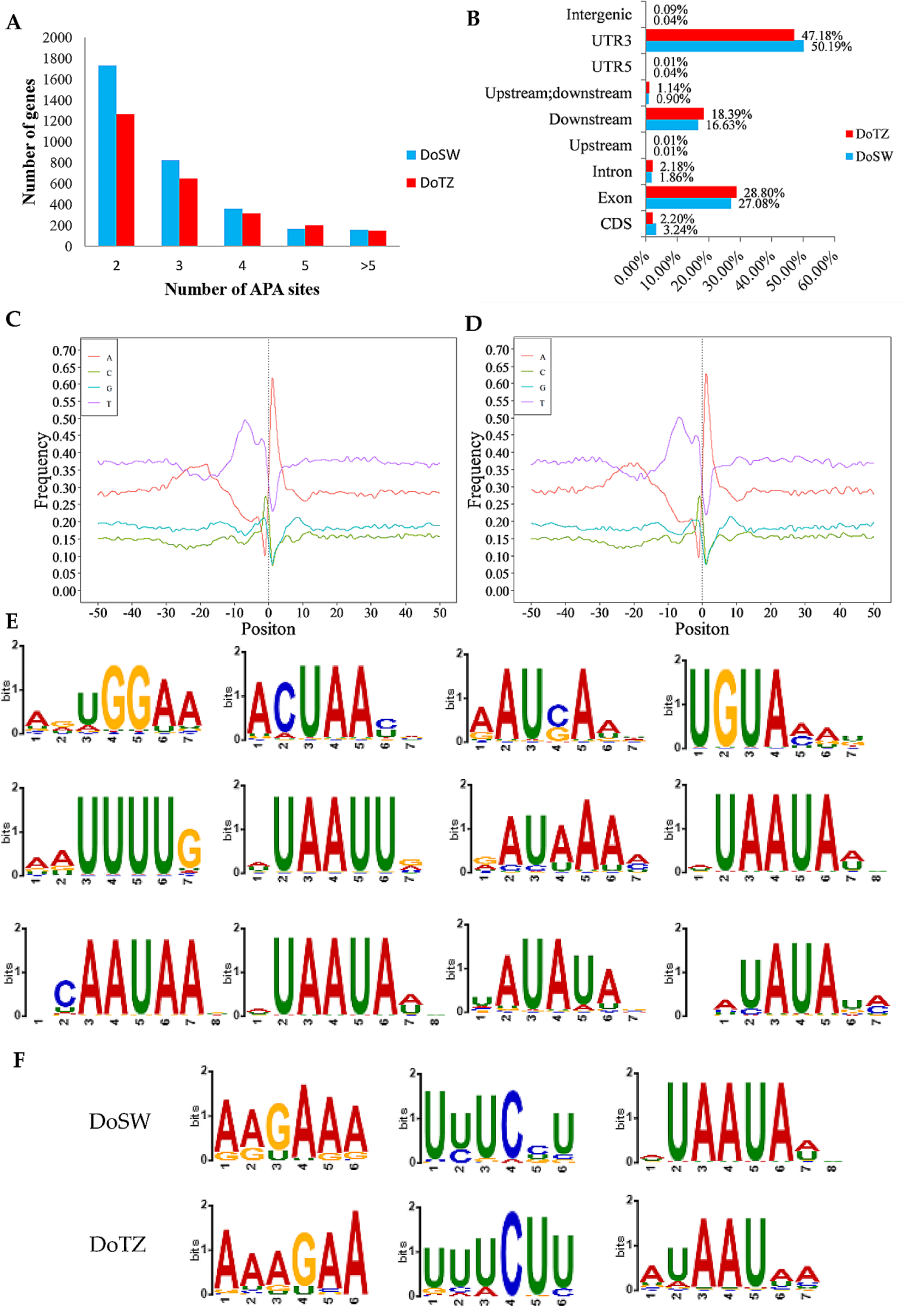
Characteristics of APA sites in *D. odorifera*. (**A**) Evaluation of the number of poly(A) sites per gene model in DoSW (sapwood) and DoTZ (transition zone) samples. (**B**) Distribution of alternative polyadenylation sites on the transcripts. The nucleotide composition in the regions surrounding the alternative polyadenylation sites in DoSW (**C**) and DoTZ (**D**) was analyzed. (**E**) Motifs shared between DoSW and DoTZ are presented. (**F**) Motifs with slight differences in DoSW and DoTZ

We applied the TAPIS [27] tool to identify APA (Alternative polyadenylation) events from Iso-Seq data and 4,164 APA genes were identified. Separately, with the help of TAPIS [27], 9,349 and 7,813 APA sites were identified from 3,244 and 2,580 genes which were detected by Iso-Seq in DoSW and DoTZ, respectively. Among the genes with APA sites, 259 genes (157 of DoSW and 146 of DoTZ) were observed with at least five poly(A) sites (Fig. 3A). On an average, 2.88 and 3.03 poly(A) sites per gene were identified from the two sample types, respectively. Most APA sites (47.2% − 50.2%) were detected in the 3’ untranslated region (UTR; Fig. 3B). Less than 1% of APA loci were distributed in the 5’ UTR and intergenic regions. In addition, about 30% of APA loci fell on exons. APA sites located upstream of the last exon could lead to changes in the expression of terminal exons, which could result in the alteration of the coding sequence of an mRNA [3]. In particular, 2.2–3.2% of APA loci fell on the annotated CDS (coding sequence) of hundreds of genes (Fig. 3B), truncating the coding sequence, and thus, providing evidences to support the notion that they have an ability to change the protein that could be coded from these genes.

Furthermore, nucleotide composition of the regions flanking the APA sites (50 nucleotides up- and down-stream) was analyzed. We observed a clear nucleotide bias for the enrichment of uracil in the upstream, and of adenine in downstream of APA sites (Fig. 3C, D). This is consistent with findings in *Sorghum bicolor* [27], *Phyllostachys edulis* [23] and *Spartina alterniflora* [4]. To determine the possibility of *cis-*elements being involved in polyadenylation, the motifs of 50 nt upstream of APA sites were investigated in all the transcripts with APA events with the help of the MEME suit. We found that 12 motifs were shared by DoSW and DoTZ (Fig. 3E), indicating that they may be important for polyadenylation. In addition, we found that some motifs were slightly different in DoSW and DoTZ, which may contribute to the occurrence of differential APA site usage of DE-APA genes in the two samples (Fig. 3F). AAUAAA serves as a canonical poly(A) signal in both, plants and animals, and it can be recognized by the cleavage and polyadenylation specificity factor (CPSF) complex, which plays a crucial role in the cleavage and polyadenylation process [3]. Similarly, UGUA motif is recognized by the cleavage stimulation factor (CstF) [3]. The presence of AAUAAA and UGUA motifs in our datasets indicated the authenticity of identified APA sites.

**Fig. 4:**
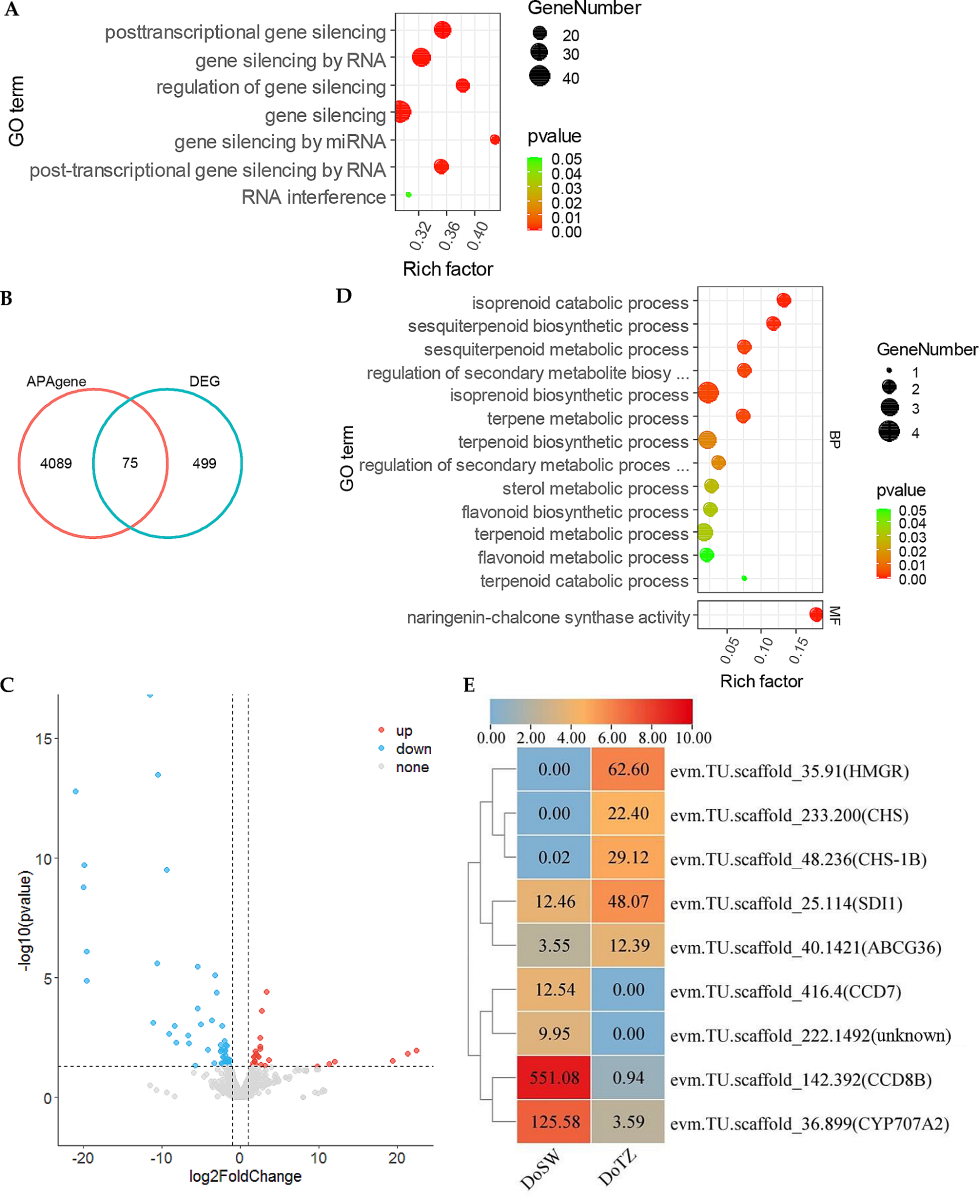
Differentially expressed APA genes involved in xylem development. (**A**) APA genes are enriched in the categories related to post-transcriptional regulatory processes. (**B**) Venn diagram of APA- and differentially expressed genes show some commonality. (**C**) Volcano plot of the significantly up- and down-regulated APA genes in the transition zone of *D. odorifera* (|log2(fold change)| ≥1, P-value < 0.05). (**D**) Differentially expressed APA genes are involved in the synthesis of terpenoids and flavonoids. (**E**) The expression profile of differentially expressed APA genes that are related to terpenoid/flavonoid metabolism. The scale bar at the top of the figure represents log2(FPKM + 1) transformed values. Original FPKM values are shown within the boxes

To better understand their functions, all APA genes identified in our research were subjected to GO (gene ontology) enrichment analysis. By using all the known reference transcripts [20] and newly identified novel transcripts as the background, our analysis indicated that APA genes were significantly involved (P-value < 0.05) in transcriptional and post-transcriptional regulatory processes (Fig. 4A, Supplementary Fig. 1), and in tissue differentiation and seedling development (Supplementary Fig. 2).

In order to investigate the potential role of APA genes in wood formation, we also investigated the expression differences of these genes across different regions of the wood. Seventy-five APA genes were differentially expressed between sapwood and transition zone (P-value < 0.05, |log2(fold change)| >1; Fig. 4B, C). Further, GO enrichment analysis revealed that multiple genes were involved in biological processes related to terpenoids and flavonoids (Fig. 4D; Supplementary Table 1), which are the characteristic components of *D. odorifera* heartwood [2].

Further analysis of these genes, enriched in terpenoid and flavonoid related GO term, was conducted by combining their GO and SwissProt annotations. We noticed that two of these genes (*CHS* and *CHS-1B*) encoded for chalcone synthase and chalcone synthase 1B. The primary product of both of these synthases is chalcone, which could, under specific conditions, spontaneously isomerize into naringenin. We also identified an *HMGR* that encodes for a 3-hydroxy-3-methylglutaryl-coenzyme A reductase, which catalyzes the synthesis of mevalonate, a specific precursor of all the plant isoprenoids. All of these three genes were highly expressed in the transition region (Fig. 4E; Supplementary Table 1), but almost not expressed in the sapwood. These results indicate a potential involvement of APA and those APA genes in determining the biochemical composition of heartwood by activities in the transition zone.

### Analysis of differential APA site usage

**Fig. 5:**
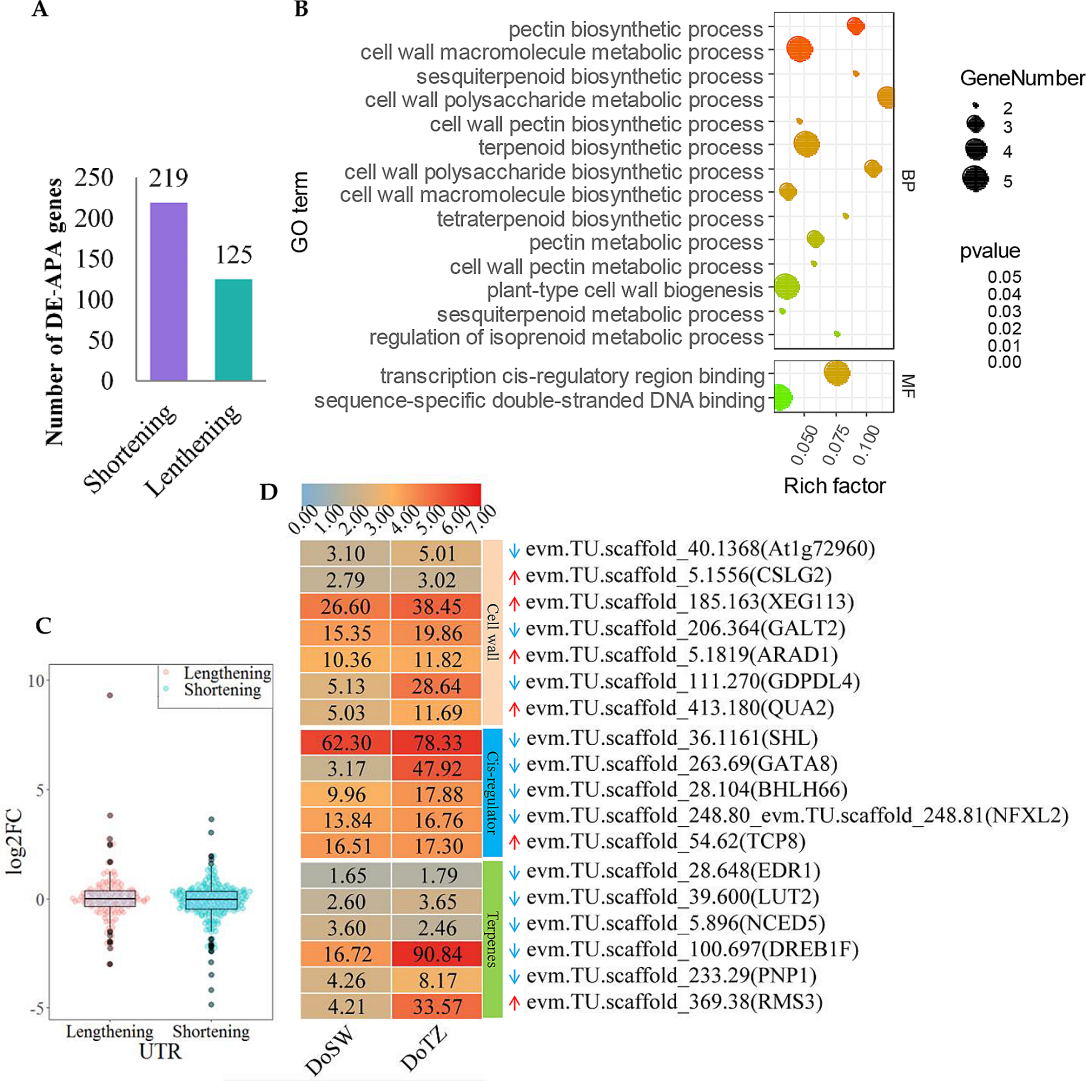
Genes with DE-APA (differential APA site usage) and involved in xylem development. (**A**) A summary of DE-APA genes identified in our analysis. The number of DE-APA genes exhibiting a shortened 3’ UTR in the transition zone is represented by violet bars, while those exhibiting a lengthened 3’ UTR are represented by green bars. (**B**) The results of GO (gene ontology) enrichment analysis showed that DE-APA genes were associated with cell wall biogenesis, terpene anabolism, and *cis-*regulation of gene transcription. The panel (**C**) shows the variation in the expression of genes with altered 3’ UTRs. The expression value of each sample is calculated as the log_2_(fold change) of FPKM between the transition zone and sapwood. FC: fold change. (**D**) shows the expression of DE-APA genes involved in cell wall synthesis, terpenoid biosynthetic and transcriptional regulation. Genes labeled with blue downward arrows have a negative r value (r < 0; these are results of APAtrap analysis), indicating that they might prefer to use shorter 3’ UTR (more proximal poly(A) site) in the transition zone as compared to the sapwood. Similarly, the genes marked with the red upward arrows have a positive r value (r > 0), indicating that they prefer to use longer 3’ UTR (more distal poly(A) site) in the transition zone than sapwood. ‘r’ represents Pearson’s product moment correlation coefficient, $\in$[-1,1]. Scale bar on the top of the picture represent log_2_(FPKM + 1) transformed values. Original FPKM values are shown within the boxes

Next, we investigated the differences in APA performance between sapwood and transition zone so that we could gain insights into their potential role in wood tissue differentiation and wood formation in *D. odorifera*. Differential poly(A) sites were quantified by combining PacBio-generated full-length reads (full-length transcript sequences) with RNA-seq technology (using Illumina platform). Percentage difference (PD) calculated by APAtrap is used to quantify the difference of APA site usage between two samples in a given gene [28]. In total, 344 genes with differential APA site usage (DE-APA) were identified (PD ≥ 0.2 and adjusted p-value < 0.05) after pair-wise comparisons of DoSW and DoTZ. These genes tend to use dissimilar lengths of 3’ UTR in different tissues by selecting diverse 3’ UTR-APA sites. When we pair-wise made comparisons of DoSW and DoTZ, we found that in transition zone, 125 DE-APA genes displayed a global 3’ UTR lengthening (PD ≥ 0.2, p-value < 0.05 and r > 0), which was attributed to the preferential utilization of distal poly(A) sites. On the other hand, 219 DE-APA genes displayed a global 3’ UTR shortening (PD ≥ 0.2, p-value < 0.05 and r < 0; Fig. 5A) as a result of the preferential usage of proximal poly(A) sites. These results indicate that a shorter 3’ UTR is more preferred in the transition region.

Enrichment analysis was performed to explore the function of DE-APA genes, which revealed their association with cell wall biogenesis, terpene anabolism, and *cis-*regulation of gene transcription (Fig. 5B; Supplementary Table 2). Three of these genes (*GATA8, BHLH66*, and *TCP8*) encode for transcription factors; *NFXL2* encodes for a NF-X1-type zinc finger protein, and *SHL* encodes for a chromatin remodeling protein.

In animals, APA regulates the stability and translation of mRNAs by changing the length of 3’ UTRs. Although seen in some studies [5, 29], it is not clear whether this phenomenon is widespread in plants. So, we wanted to know whether the global mRNA accumulation of DE-APA genes was significantly related to their global 3’ UTR shortening or lengthening. First, we surveyed the expression levels of 344 DE-APA genes with longer or shorter 3’ UTR expressions in the sapwood. Overall, we found little difference in the expression of these genes, regardless of whether they expressed lengthened or shortened 3’ UTRs (Fig. 5C). Further, we investigated the expression profiles of 18 genes that were related to either the synthesis or metabolism of terpenes and cell wall components, or those enriched in the processes of ‘transcription *cis-*regulatory region binding’ and ‘sequence-specific double-stranded DNA binding’. Most of these genes exhibited higher expression levels in the transition zone regardless of whether their 3’ UTRs tend to be longer or shorter in this zone (Fig. 5D). These findings indicate that the 3’ UTRs lengthening in the DoTZ does not act as a universal mechanism for regulating mRNA accumulation.

### Analysis of alternative- and differential-alternative splicing

To investigate the genes that may have undergone a differential alternative splicing (DAS), we used the SUPPA2 software [30] to identifty DAS events. We detected a total of 3,084 AS genes from the DoSW (1,555) and DoTZ (1,486) samples, respectively. AS genes produced 11,028 splicing events, which generated 5,598 transcript isoforms (10,845 in DoSW and 9,235 in DoTZ, respectively). In total, 2,636 genes were detected with multiple spliced isoforms, with an average of about 1.82 transcript isoforms per gene. This result is an improvement on the existing genome annotation, in which only one transcript for a gene is reported. Father, it was evident that isoforms were more abundant in DoTZ than DoSW (Fig. 2A). Among the predicted multi-isoform AS genes, 58.18 − 65.60% genes coded for two isoforms, 19.23 − 20.22% coded for three; for the remaining AS genes, we noticed that their percentages progressively decreased as the number of associated isoforms increased (Fig. 2C). In particular, our Iso-Seq sequencing detected two genes with obvious multi variable splicing transcripts in DoTZ samples: One of these, *CALS2*, encoding for a callose synthase 2, had 45 splicing transcripts in DoTZ samples, while only 10 transcripts in DoSW samples. *CALS2* is involved in callose synthesis at the forming cell plate during cytokinesis. For the other gene, evm.TU.scaffold_36.141, potentially encoding for a Glutamate synthase [NADH], 39 transcripts were detected in DoTZ, while none was detected in DoSW (Supplementary Fig. 3). This enzyme is required for the assimilation of symbiotically fixed nitrogen into amino acids in root nodules.

**Fig. 6:**
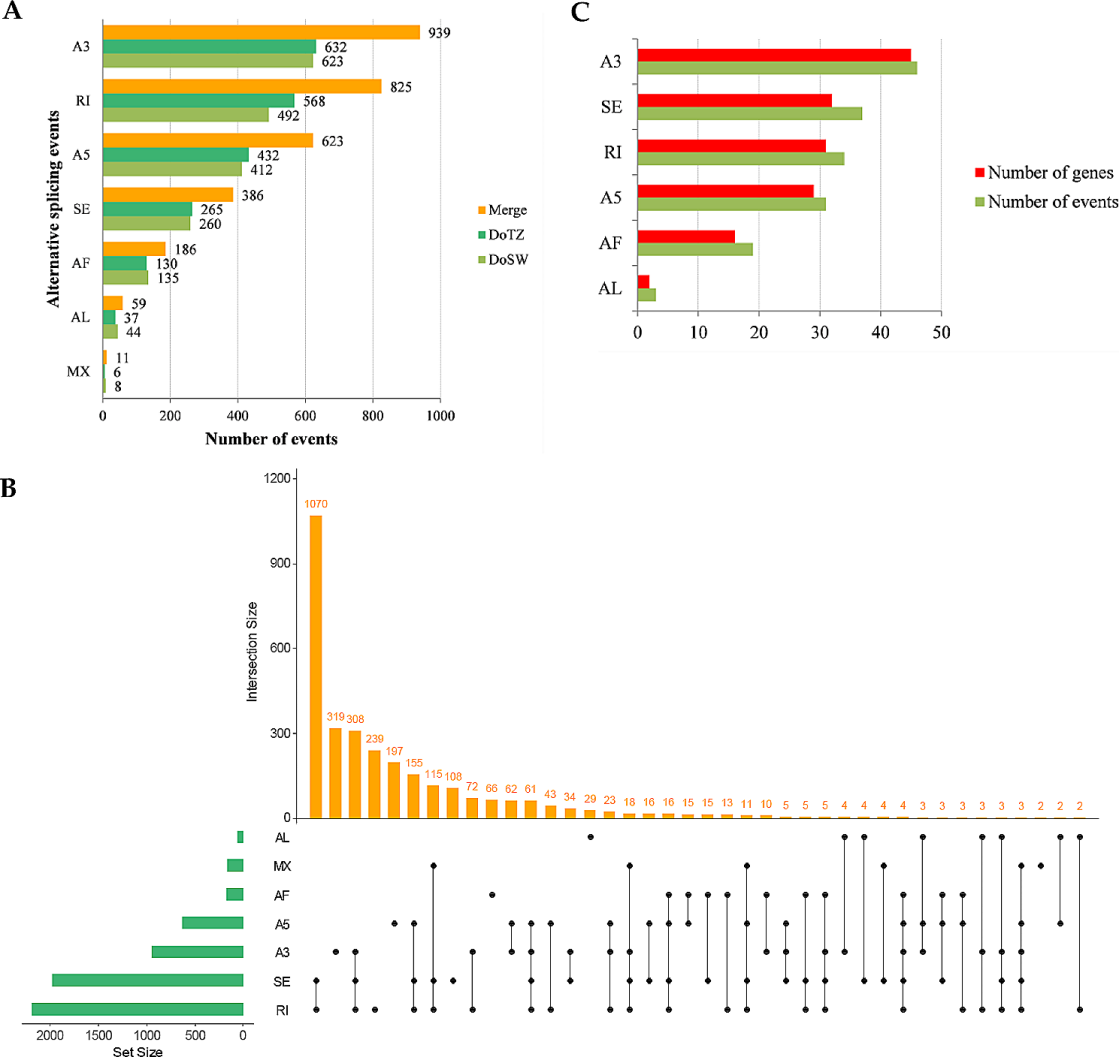
Characterization of the AS (alternative splicing) event in *D. odorifera*. (**A**) The number of each type of AS events detected in sapwood (DoSW) and transition zone (DoTZ) by SUPPA2. (**B**) The Upset plot shows a genome-wide distribution of AS events in AS-related genes in *D. odorifera*. The bar chart in the upper panel shows the gene counts for each group/category. The bar chart located at the bottom left indicates the gene count for each of the specific type of AS event. The dotted line on the bottom right displays the types of events present within a group. (**C**) The overall count of occurrence of AS events in DAS (differential alternative splicing) genes as compared to the total number of DAS genes that experienced specific types of AS events

The AS events are classified into seven major types: RI (retained intron), SE (skipping exon), MX (mutually exclusive exons), A3 (alternative 3’ splice sites), A5 (alternative 5’ splice sites), AF (alternative first exons) and AL (alternative last exons) [30]. Among these, A3 events were the most prominent type in our investigation, and accounted for 31.00% (939). This was followed by RI (825, 27.24%), A5 (623, 20.57%), SE (386, 12.74%), AF (186, 6.14%), AL (59, 1.95%) and MX (11, 0.36%) (Fig. 6A). However, except for AF, AL, and MX, the other four events (A3, A5, RI and SE) appeared more in DoTZ than in DoSW (Fig. 6A). Additionally, genome-wide AS events were also identified, which showed that RI was a dominant splicing mode. Further, we found that 10 AS genes were exposed to five events, and 117 genes participated in four types of AS events. Similarly, 630 and 1,367 genes were engaged in three or two AS events, respectively. The remaining 960 genes had experienced only one AS event (Fig. 6B).

**Fig. 7:**
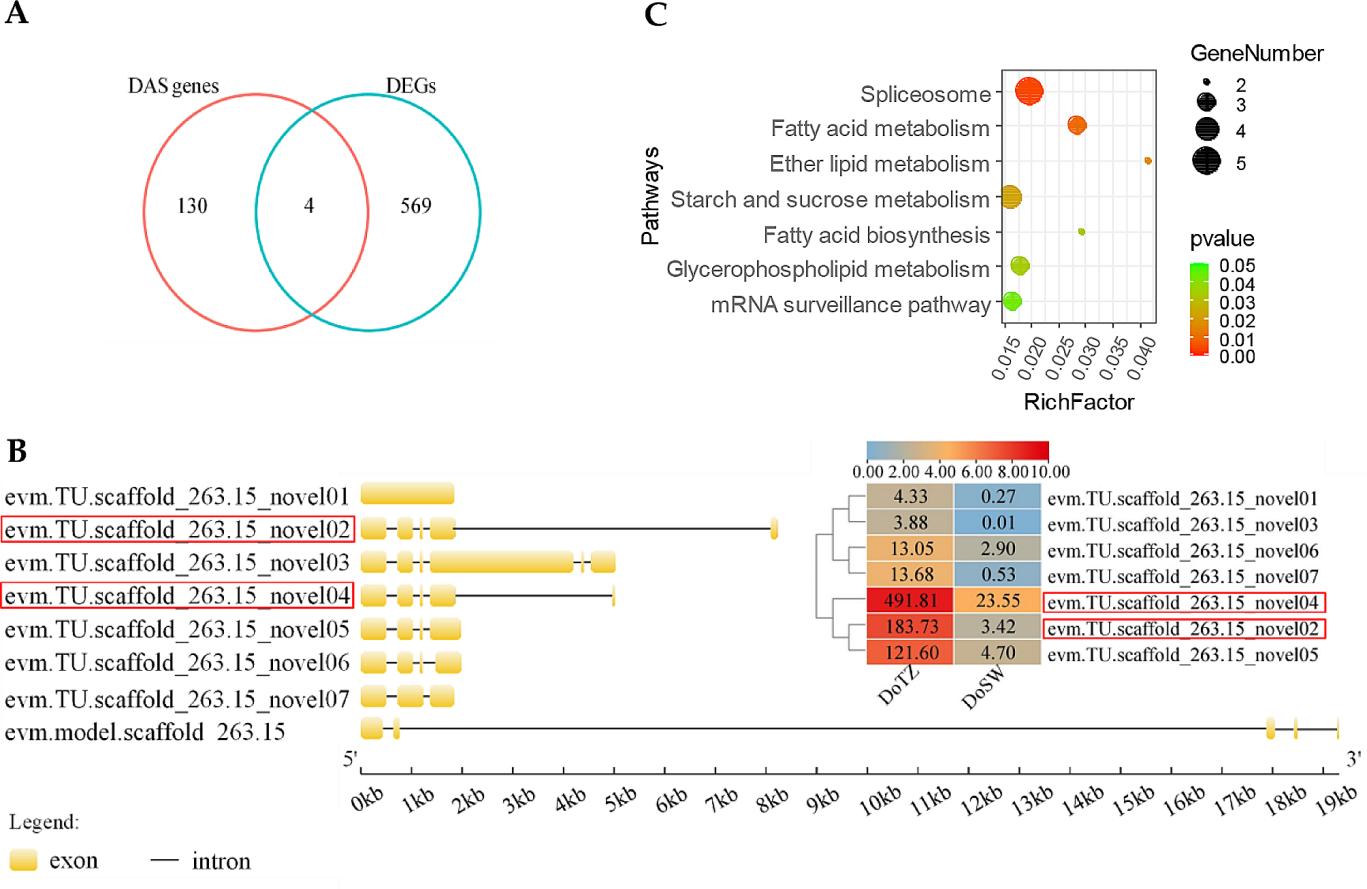
Differential alternative splicing analysis. (**A**) Venn diagram shows the comparison between the number of DAS genes and the number of differentially expressed DAS genes. (**B**) The splicing isoforms of the gene evm.TU.scaffold_263.15. The bottom left of the picture shows the transcript structure of the gene. The upper right panel shows the heat map of expression of all the spliced transcripts of this gene. Scale bar on the top of panel represent log2(FPKM + 1) transformed values. Original FPKM values are also shown within the cells. The red box marks two transcripts that have experienced differential alternative last exon (AL) splicing. (**C**) The most significantly enriched KEGG (Kyoto Encyclopedia of Genes and Genomes) pathways for DAS genes

To explore the differential AS landscape in *D. odorifera* between the DoSW and DoTZ tissues, the “diffSplice” subcommand of SUPPA2 was utilized. DAS (differential alternative splicing) events were identified on the basis of difference of percentage or proportion of spliced-in values (ΔPSI) using the criteria of P-value < 0.05 and |PSI| >0.1. In total, 170 DAS events were identified between DoSW and DoTZ zones from 134 genes. A3 was the main subtype among the splicing events at transcriptome level (Fig. 6A, C). It is possible that A3 may be playing a special role in regulating gene expression in the xylem of *D. odorifera*. To assess the number of genes that were both, differentially expressed and subjected to DAS, a comparative analysis was conducted between the DEGs and DAS events, and following four DEGs were identified (Fig. 7A; Supplementary Table 3): *FRO8* (ferric reduction oxidase 8), *PHT1-7* (probable inorganic phosphate transporter 1–7), *LAX1* (auxin transporter-like protein 1) and *Novelgene0421* (Agglutinin-2) (|log2(fold change)| >1 and P-value < 0.05; Supplementary Table 3). We also found several DAS genes as encoding for transcription factor, such *BZIP16* and *MYB59*. We noticed that a isoliquiritigenin 2’-O-methyltransferase (evm.TU.scaffold_263.15; ΔPSI = 0.2, P-value = 0.02) and wall-associated receptor kinase-like 14 (*WAKL14*) experienced differential alternative splicing. Noticeably, evm.TU.scaffold_263.15 has an extremely high expression (up to 24 times) in the transition zone as compared to the sapwood (log2(fold change) (DoTZ vs. DoSW) = 4.613). It experienced differential alternative last exon (AL) splicing, thus generating evm.TU.scaffold_263.15_novel02 and evm.TU.scaffold_263.15_novel04, while the later had extremely high expression level (Fig. 7B).

The GO enrichment analysis of DAS genes showed that they were associated with 41 biological processes, such as regulation of mRNA splicing via spliceosome, and regulation of mRNA processing, 9 cellular component such as transcription regulator complex and spliceosomal complex, and 9 molecular functions, such as RNA helicase activity, ATP-dependent activity, and acting on RNA (Supplementary Fig. 4). Further, the KEGG enrichment analysis of DAS genes showed that they were associated with pathways of spliceosome, fatty acid metabolism, ether lipid metabolism, starch and sucrose metabolism, fatty acid biosynthesis, glycerophospholipid metabolism and mRNA surveillance pathways (Fig. 7C).

### Long noncoding RNA identification

**Fig. 8:**
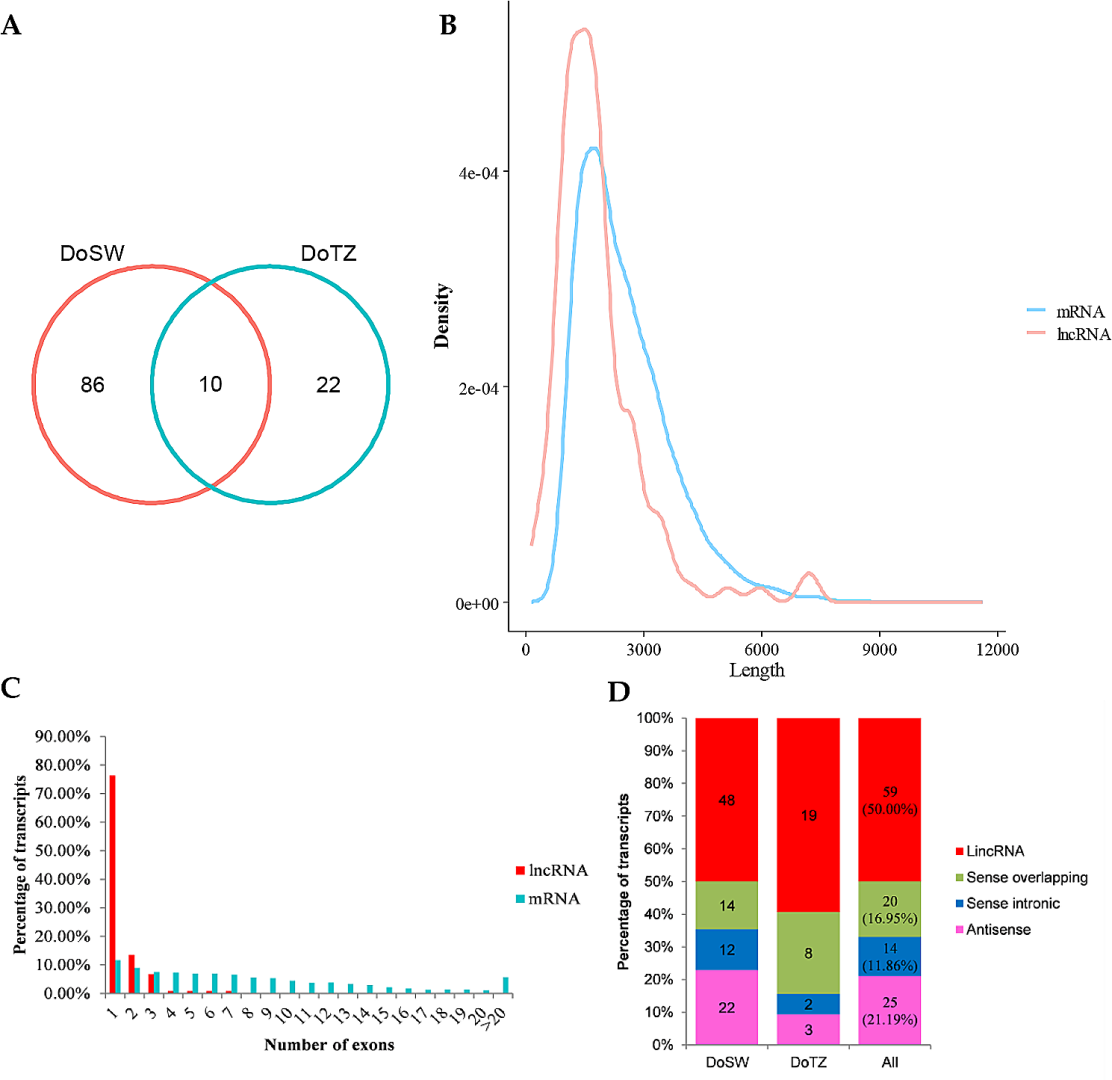
*D. odorifera* lncRNAs, identified by Iso-Seq. (**A**) Venn diagram shows the number of shared and distinct lncRNAs identified from sapwood (DoSW) and transition zone (DoTZ). (**B**) Transcript size distributions for lncRNAs as compared to those for protein-coding transcripts. (**C**) The number of exons per transcript for lncRNAs and protein-coding transcripts. (**D**) Distribution of different types of lncRNAs in sapwood and transition zone of *D. odorifera*

Long non-coding RNAs (lncRNAs) play a significant role in regulating a gene’s expression at multiple levels, including during transcription, splicing, and translation [31]. However, limited information is available about lncRNAs in *D. odorifera*. We used four computational approaches (described in the Methods section) to identify lncRNAs from the Iso-Seq isoforms. Finally, we obtained a total of 118 highly reliable lncRNAs (96 and 32 from DoSW and DoTZ, respectively). Only 10 lncRNAs were shared by the two tissues (Fig. 8A).

The length of identified lncRNAs ranged from 217 to 7,240 nt, with a majority (98, > 83%) being ≥ 1,000 nt. The average length of lncRNAs was 1,826 nt, which was much shorter than the mean length of all 14,938 isoforms (2,507 nt) detected by Iso-Seq (Fig. 8B). In *D. odorifera*, compared to mRNAs, which have an average of 8.24 exons, lncRNAs only have an average of 1.42 exons (Fig. 8C). *D. odorifera* lncRNAs were categorized into 4 groups: 59 (50.00%) lincRNA, 25 (21.19%) antisense, 14 (11.86%) sense intronic and 20 (16.95%) sense overlapping lncRNAs (Fig. 8D).

### Prediction of targets of lncRNAs in the protein-coding genes

**Fig. 9:**
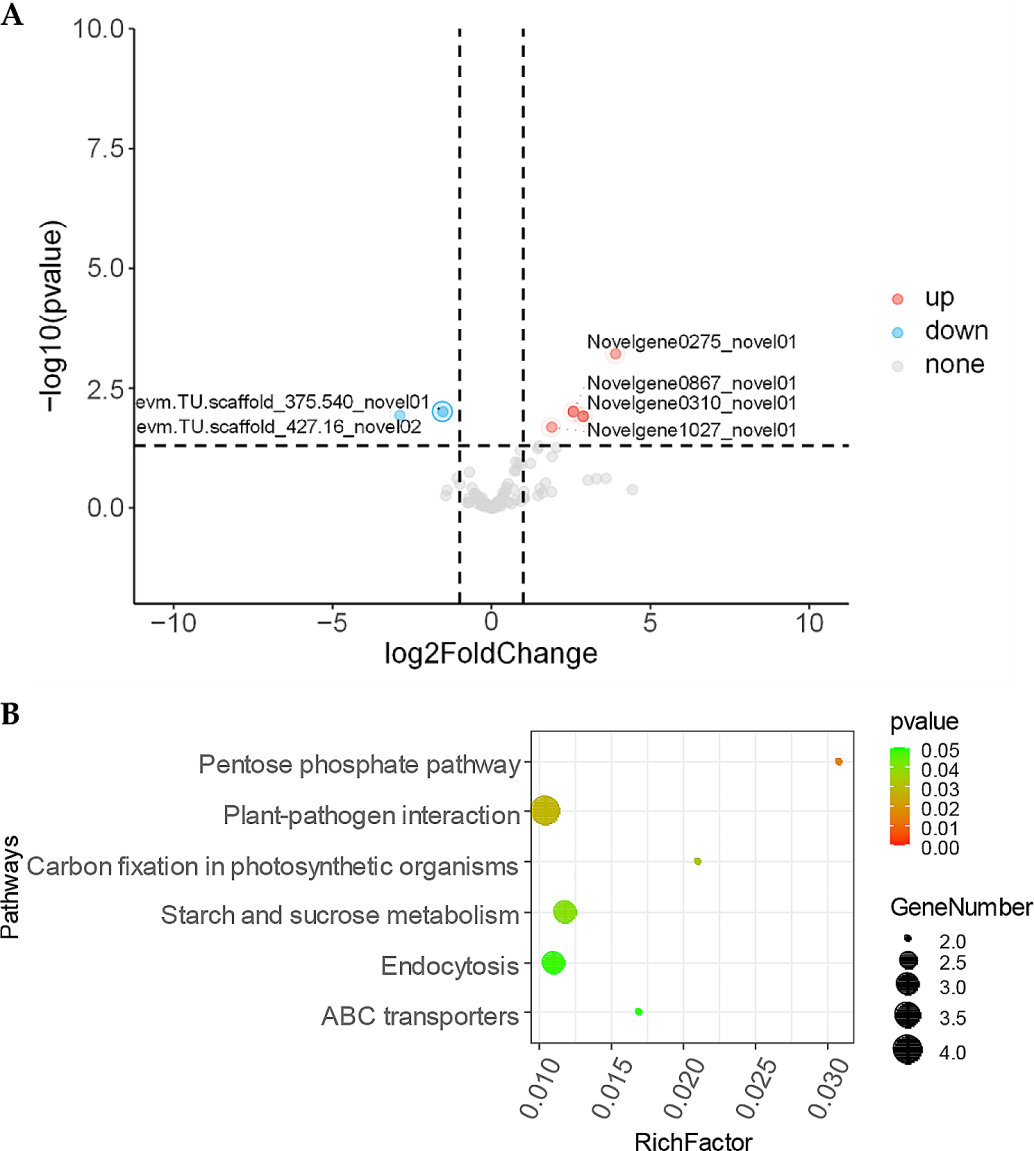
Differentially accumulated lncRNAs possibly involved in xylem development. (**A**) Volcano plot of the significantly up- and down-regulated lncRNAs in the in transition zone (|log2(fold change)| ≥1, P-value < 0.05). (**B**) The most significantly enriched KEGG (Kyoto Encyclopedia of Genes and Genomes) pathways for the trans-targeted genes of differently accumulated lncRNAs are presented

To study the changes in patterns of lncRNAs during wood development, the accumulation levels of all lncRNA in the DoSW and DoTZ were compared using Cuffdiff program [32]. There were 6 differentially accumulated lncRNAs (|log2(fold change)| >1 and P-value < 0.05), of which 4 were up- and 2 were down-regulated (Fig. 9A). The differential accumulation of lncRNA in sapwood and transition zone indicated that they may regulate the development and differentiation of xylem tissue and could play a role in gene expression by interacting with target genes. So, the potential target genes of 6 differentially accumulated lncRNAs were predicted in terms of their regulatory effects on their targets (*cis-* or *trans*- acting).

We determined potential *cis-*regulated target genes of lncRNAs by searching the protein-coding genes located in close proximity (within a genomic window of 100 kb) of a lncRNA. Six lncRNAs could potentially have *cis-*regulatory effects on 110 mRNAs (110 lncRNA-target pairs). Additionally, a Pearson’s correlation coefficient of the accumulation of lncRNAs and their potential target gene pairs was calculated. The accumulation of 3 lncRNAs and their corresponding 16 target mRNAs (corresponding to 15 genes) were strongly correlated (|Pearson’s correlation coefficient| >0.8, P-value < 0.05; in 16 target-lncRNA pairs; Supplementary Table 4). These 15 genes are considered as highly credible targets and their functions were explored by checking their GO terms. We found that these *cis-*target genes participate in the biological processes such as RNA splicing, regulation of programmed cell death and flavonoid biosynthetic process, as well as could have the molecular functions of single-stranded RNA binding, protein prenyltransferase activity and naringenin-chalcone synthase activity (Table 2).

**Table 2:** The GO (gene ontology) information of *cis-*regulated target genes involved in isoprene and flavonoid biosynthesis

Gene ID	Gene Name	GO	Description	Level
evm.TU.scaffold_5.712	*CHS1*	GO:0016210	naringenin-chalcone synthase activity	molecular function
evm.TU.scaffold_5.712	*CHS1*	GO:0009813	flavonoid biosynthetic process	biological process
evm.TU.scaffold_5.712	*CHS1*	GO:0071704	organic substance metabolic process	biological process
evm.TU.scaffold_5.712	*CHS1*	GO:0009812	flavonoid metabolic process	biological process
evm.TU.scaffold_5.709	*RGTB2*	GO:0008318	protein prenyltransferase activity	molecular function
evm.TU.scaffold_5.709	*RGTB2*	GO:0097354	prenylation	biological process
evm.TU.scaffold_5.709	*RGTB2*	GO:0099402	plant organ development	biological process
evm.TU.scaffold_5.709	*RGTB2*	GO:0042981	regulation of apoptotic process	biological process
evm.TU.scaffold_5.709	*RGTB2*	GO:0043067	regulation of programmed cell death	biological process
evm.TU.scaffold_427.20	*MTERF4*	GO:0003727	single-stranded RNA binding	molecular function
evm.TU.scaffold_427.20	*MTERF4*	GO:0090304	nucleic acid metabolic process	biological process
evm.TU.scaffold_427.20	*MTERF4*	GO:0008380	RNA splicing	biological process
evm.TU.scaffold_427.20	*MTERF4*	GO:0006725	cellular aromatic compound metabolic process	biological process
evm.TU.scaffold_427.20	*MTERF4*	GO:0019438	aromatic compound biosynthetic process	biological process

The prediction of potentially *trans*-regulated lncRNA targets was conducted with the help of LncTar [33]. Six lncRNAs showing differential accumulations were predicted to potentially interact with 2,679 mRNAs in a *trans*-regulatory manner (ndG ≤-0.1). Expression correlation analysis was conducted to obtain highly credible *trans*-regulated target genes. Six differentially accumulating lncRNAs were highly correlated with 151 potential target mRNAs (|Pearson’s correlation coefficient| >0.8, P-value < 0.05) in 155 lncRNA-target pairs, in which 135 pairs were positively correlated (Pearson’s correlation coefficient > 0), while 20 were negatively correlated (Pearson’s correlation coefficient < 0; Supplementary Table 5). The KEGG enrichment results showed that the *trans*-regulated targets of lncRNAs could play an important role in carbon fixation and plant-pathogen interaction (Fig. 9B).

### Validation of gene expression by RT-qPCR

To validate the reliability of the RNA-seq data, the expression patterns of six genes were detected by RT-qPCR (Supplementary Table 6). The expression patterns of all of these genes in the DoSW and DoTZ showed similar trends between the RNA-seq analysis and RT-qPCR analysis (Supplementary Fig. 5). Although the relative expression level calculated by RNA-sequencing data did not exactly match that detected by RT-qPCR, their trend is consistent for the genes tested, demonstrating the reliability of the sequencing data.

## Discussion

### APA is involved in RNA processing and wood formation including the synthesis of heartwood substances of *D. odorifera*

The use of an APA site could alter the coding capacity by truncating the upstream region of the terminal exon or by the inclusion/exclusion of key sequence elements for mRNA stability, location, and translation regulation [3, 34, 35]. Results of our GO enrichment analysis of all the APA genes were consistent with this principle. A considerable number of APA genes were enriched in function associated with regulation of transcription, RNA splicing, RNA-3’ end processing, regulation of RNA stability, RNA localization, signal sequence binding, mRNA cap binding complex and mRNA 3’-UTR binding (Supplementary Fig. 1). In addition, many genes were enriched in biological processes related to gene silencing. Especially, tens of genes were enriched in the silencing process mediated by miRNA (Fig. 4A), indicating that APA could mediate complex post-transcriptional regulation.

Studies in humans have shown that APA can participate in the regulation of processes related to health and disease [36]. Similarly, studies in plants have also shown that APA can participate in regulating gene expression in response to stresses, and flowering time [35, 37]. However, studies on the role of APA in the formation of wood of the trees are still highly limited. Here, we quantitatively profiled the APA sites usage between sapwood and transition zone of *D. odorifera*. In the GO functional enrichment analysis of all identified APA genes, we observed that they were not only enriched in functions related to RNA processing, but also in functions related to tissue differentiation and seed development (Supplementary Fig. 2). This indicates that APA could play an important role in the growth and development in *D. odorifera*.

More importantly, we identified 75 APA genes with significant differences in their expression levels between sapwood and transition region. Enrichment analysis of their GO terms indicated that some of these APA genes were related to terpenoid and flavonoid metabolism (Fig. 4D). Two of these (*CHS* and *CHS-1B*) encoded for chalcone synthase and chalcone synthase 1B, whereas one of them (*HMGR*) encoded for a 3-hydroxy-3-methylglutaryl-coenzyme A reductase. They were highly expressed in the transition zone, but hardly in the sapwood (Fig. 4E). Chalcone synthase can catalyze the synthesis of 4,2’,4’,6’-tetrahydroxychalcone (also termed naringenin-chalcone or chalcone), which, under specific conditions, can spontaneously isomerize into naringenin [38]. Both, chalcone and naringenin, are the components of the heartwood of *D. odorifera* [39, 40]. Further, 3-hydroxy-3-methylglutaryl-coenzyme A reductase catalyzes the synthesis of mevalonate, which is a specific precursor of all the isoprenoid compounds in plants [38, 41], as the isoprene units make up the terpenes [42, 43]. Flavonoids and terpenoids are the main secondary metabolites in the heartwood of *D. odorifera* [2]; flavonoids are considered to play a critical role in the color formation in mahogany heartwood [19, 40]. Terpenoids are also an important source of fragrance in *D. odorifera* [44, 45]. Thompson et al. (2006) found that the highest concentrations of terpenes were in heartwood of loblolly pine (*Pinus taeda*), while their lowest levels were recorded in the outer sapwood; the inner sapwood had moderate terpene levels. The high expression level of the three APA genes, which encoded for chalcone synthase, chalcone synthase 1B, and 3-hydroxy-3-methylglutaryl-coenzyme A reductase, indicates the importance of the transition region in the synthesis of heartwood substances in *D. odorifera*, where APA phenomenon might play an essential role in the production of these substances. However, the mechanism of APA in this process still needs further research.

To investigate the regulatory role of differential expression of APA, we compared the differences in APA usage patterns between the sapwood and the transition zone within the xylem of *D. odorifera*. In our GO enrichment analysis of 344 DE-APA genes, we found that the biological process of cell wall macromolecule biosynthesis/metabolism, and the biosynthesis of cell wall pectin were enriched (Fig. 5B). The wood fiber is formed from several compounds that include lignin, cellulose, hemicellulose and a certain amount of pectin [46]. Pectin, a carbohydrate polymer, exists in the middle layer of a plant’s adjacent cell wall along with cellulose, and it binds the cells or tissue together [47]. Plant cell walls consist of an intricate polymeric matrix comprising of lignin, cellulose, hemicellulose, pectin, and glycoproteins. Non-cellulosic cell wall polysaccharides significantly influence several physical properties of plant cell walls, such as tensile strength, crystallinity and density of plant cell walls [46], which in turn affect qualities such as density and toughness of a wood. Wood formation includes biosynthesis of cell wall macromolecules, cell division, cell expansion, secondary cell wall deposition, lignification and programmed cell death. Our results suggest a potential regulatory role of APA in the wood formation of *D. odorifera*. We also found that APA bias events might have occurred in genes involved in terpenoid biosynthetic or metabolic process in xylem (Fig. 5B). This is another clue for the involvement of APA in regulating the synthesis of heartwood substances of *D. odorifera*.

### Alternative splicing participates in regulating the synthesis of heartwood substances of *D. odorifera*

Several studies have demonstrated a significant involvement of AS in various aspects of plant development and stress tolerance [48–51], but it has not yet been characterized in *D. odorifera*. In this study, we detected 11,028 AS events from 8,951 genes (Fig. 6A), corresponding to 5,598 splice isoforms. Further, splice isoforms of 2,124 genes were derived from combination of multiple types of events (Fig. 6B). These results suggest that AS increases the transcriptome complexity in the xylem of *D. odorifera*.

We further quantified the AS events in the sapwood and the transition zone to study differences in AS-usage across the two xylem regions. We could identify 170 DAS events from 134 genes. For example, evm.TU.scaffold_263.15, which encodes for isoliquiritigenin 2’-O-methyltransferase, expressed 24 times higher in transition zone as compared to the sapwood and encountered a differential alternative last exon (AL) splicing (Fig. 7B). It produced two splicing isoforms, evm.TU.scaffold_263.15_novel02 and evm.TU.scaffold_263.15_novel04. Isoliquiritigenin 2’-O-methyl transferase can catalyze the reaction between isoliquiritigenin and S-adenosine-L-methionine to produce 2’-O-methylisoliquiritigenin [52], thus affecting the accumulation of isoliquiritigenin and 2’-O-methylisoliquiritigenin. Isoliquiritigenin and 2’-O-methylisoliquiritigenin are chalcones contained in *D. odorifera*, which are reported flavonoids of heartwood components [53, 54]. Isoliquiritigenin from the heartwood of *D. odorifera* has anti-inflammatory properties [55], whereas 2’-O-methylisoliquiritigenin has strong cytotoxic activity against the three types of human cancer cells (A-549, SK-MEL-2 and SK-OV-3) [56]. Therefore, these two substances have important medical value, and we were able to determine the genes that could play a role in their accumulation in heartwood of *D. odorifera*. We further found that evm.TU.scaffold_263.15_novel04 has a relatively higher expression than evm.TU.scaffold_263.15_novel02, the other splicing isoform produced by alternative last exon, in both transition zone (fold change = 2.68) and sapwood (fold change = 6.88) (Fig. 7B). This further indicates the regulation potential of alternative splicing during the accumulation of chalcones. The identification of such genes not only contribute towards elucidating the mechanism of synthesis of wood components, it also has potential value in the studying the biosynthesis of important drugs.

Comparative analysis of AS events between the sapwood and the transition zone also revealed the transcription factors that experienced DAS. Our data indicates that AS might be highly relevant for xylem development in *D. odorifera*, whose specific mechanism needs elucidation in the future studies.

### LncRNAs are involved in regulating the synthesis of secondary metabolites in the xylem of *D. odorifera*

In recent years, lncRNAs have been reported as important regulators in post-transcriptional processes in plants [31, 57, 58]. They are also involved in the regulation of wood formation [59–61], however, their identity and role in *D. odorifera* still remains unknown. In this study, 118 highly reliable lncRNAs were systematically identified in the xylem of *D. odorifera* by Iso-Seq. We identified six differentially expressed lncRNAs (Fig. 8A) that might play regulatory roles in the development and differentiation of xylem tissue in *D. odorifera*.

LncRNAs have the capacity to regulate their target genes’ expression through both, *cis*- and *trans*- mechanisms [13]. In this study, a group of strongly co-expressed lncRNA-target pairs were found, which included several *cis*-targets related to single-stranded RNA binding, protein prenyltransferase activity and naringenin-chalcone synthase activity (Table 2). We noticed that *CHS1* encodes chalcone synthase 1, which, like chalcone synthase and chalcone synthase 1B encoded by *CHS* and *CHS1B*, also has a chalcone synthase activity. *CHS1* was identified as a *cis*-regulated target of a lncRNA, Novelgene0275_novel01. Similarly, *RGTB2* was identified as a *cis*-regulated target of evm.TU.scaffold_5.709_novel01 lncRNA. RGTB2 encodes geranylgeranyl transferase type-2 subunit beta 2, has a protein prenyltransferase activity, and might participate in the biological process of prenylation. Prenylation has a potential to affect the synthesis, metabolism or accumulation of isoprene in plant cells, which could affect the synthesis of terpenes. Further, a lncRNA, evm.TU.scaffold_427.16_novel02, was predicted to be able to *cis*-regulate *MTERF4* by interact with its mRNA; *MTERF4* encodes a transcription termination factor that binds single-stranded RNA and could participate in biosynthesis of aromatic compounds. Together, these findings collectively indicated lncRNAs’ role in the accumulation of terpenes in xylem.

## Materials and methods

### Plant samples

A *D. odorifera* tree, used for experiments in this study was approximately 7 years old, and were cultivated in an artificial nursery located in Hainan Province, China (19°38′56″N, 110°14′29″E), during the normal growing season without any treatments. This tree had already formed the heartwood (Supplementary Fig. 6). We obtained sampling permission before sampling our experiment materials. A sample of sapwood was collected from the xylem tissues in close proximity to the cambium, and a transition zone was sampled from the xylem near the heartwood, following a previously described protocol [62]. After collection, the samples were promptly immersed in liquid nitrogen, transported to the laboratory and stored at -80 °C in an ultra-low freezer until further experiments.

### PacBio library preparation, sequencing and data analysis

Total RNA from all the samples was extracted by the CTAB method. RNA degradation and contamination was assessed on 2% agarose gels, the purity was determined with the help of a micro-spectrophotometer and RNA-integrity was evaluated with the help of RNA Nano 6000 Assay Kit of the Agilent Bioanalyzer 2100 system (Agilent Technologies, CA, United States). RNA samples with a minimum RNA integrity number (RIN) of 7.0 were selected for library construction and Iso-Seq sequencing. The Iso-Seq library was prepared according to the Isoform Sequencing protocol (PN100-092-800-03). The sequencing was then conducted by PacBio Sequel II System at Novogene Bioinformatics Technology Co., Ltd. (Beijing, China).

PacBio SMRT Link v6.0 software was used for Iso-Seq data analysis. The raw sequencing data was processed to obtain the subread sequences. Circular consensus sequences (CCSs) were generated by performing error correction on the subreads. These CCS reads were subsequently categorized into full-length and non-full length reads on the basis of the presence of 5′ primer, 3′ primer, and the poly(A) tail within the sequence. Among the full-length reads, those that did not include any additional copies of the adapter sequence were categorized as full-length non-chimerics (FLNCs). The FLNC sequences were then clustered with the help of iterative clustering and error correction (ICE) algorithm (PacBio SMRT Link v6.0) to generate the clustered consensus sequences. These cluster consensus sequences were further corrected with the help of non-full-length sequences by Arrow (PacBio SMRT Link v6.0) to obtain the polished consensus sequences. Finally, polished consensus sequences were corrected using Illumina data with LoRDEC [63].

### Illumina datasets and their analysis

Datasets of Illumina sequencing were obtained from our prior investigation on the expression patterns of miRNAs and their potential regulatory role in the process of xylem differentiation in *D. odorifera* [64]. They were generated from six samples of sapwood and transition zone from three trees. Raw RNA-seq data sets (accession numbers: CRA006117) can be obtained from the Genome Sequence Archive [23] in BIG Data Center (BIG Data Center Members, 2019), Beijing Institute of Genomics (BIG), Chinese Academy of Sciences.

The raw data (raw reads) was initially processed with the help of Fastp [65] for read trimming, data-filtering and read-level quality control. In this step, clean sequences were obtained by removing the adapter- and poly-N-containing reads as well as those with low-quality scores. The mapping of these clean reads to the reference genome sequence was conducted by HISAT2 [66]. Gene expression levels were evaluated with FPKM (fragments per kilobase of transcript per million fragments mapped) values, which were computed with the help of Stringtie 2.1.2 [67]. The differential expression of genes between two groups was conducted with DESeq2 [68], with a cutoff P-value ≤ 0.05 and a fold change ≥ 2.

### Novel gene identification and alternative polyadenylation analysis

Unannotated genes and alternative polyadenylation sites were identified with the help of TAPIS software [27]. MEME-ChIP [69] analysis was performed with the help of website of MEME Suite 5.5.0, to identify the poly(A) sequence signals, after the sequence of 50 nt upstream of the poly(A) sites were extracted.

APAtrap [28] was employed to identify genes with significant APA dynamics. First, the “identifydistal3UTR” module of APAtrap was run to refine the annotated 3′ UTRs and to identify a new 3’ UTR or 3’ UTR extension. Second, the “predictAPA” module of APAtrap was implemented to infer all possible APA sites and to estimate their corresponding usage. Third, the “DE-APA” module was used to detect genes with significant changes in the usage of APA sites between two samples. Percentage difference (PD) was used to quantify the difference of APA site usage in a given gene. Genes with significant differential APA site usage (PD ≥ 0.2 and adjusted p-value < 0.05) were designated as DE-APA genes (genes with differential APA site usage).

### Characterization of AS events

SUPPA2 [30] was used to identify the AS events, which were classified into seven major types by AS codes: RI (retained intron), SE (skipped exon), MX (mutually exclusive exon), A5 (alternative 5’ splice site), A3 (alternative 3’ splice site), AF (alternative first exon) and AL (alternative last exon). The inclusion parameter percentage or proportion spliced-in (PSI) values for each AS event was calculated from the TPM (transcripts per kilobase per million mapped reads) values with the help of SUPPA2-psiPerEvent module of SUPPA2. Differential expressions of the alternative slicing (DAS) were determined on the basis of difference of percentage or proportion spliced-in (ΔPSI) values between the samples with the help of diffSplice module of SUPPA2, and events with P-value < 0.05 and |ΔPSI| ≥0.1 were retained as DAS events while the related genes were termed as DAS genes.

### Functional annotation and enrichment analysis

The eggNOG 5.0 database [70] was used to obtain GO (Gene Ontology) terms and KEGG (Kyoto Encyclopedia of Genes and Genomes) pathway information of genes. Coding sequences were submitted to eggNOG-mapper v2 to summarize functional information of genes. Sequences that were not assigned KEGG terms by eggNOG-mapper were submitted to KAAS (KEGG automatic annotation server) [71] to obtain a comprehensive assignment of genes to pathways. A final KEGG pathway annotation file was generated by combining the output of eggNOG-mapper and KAAS. Transcripts were further annotated through BLASTX searches against Swiss-Prot database, with an e-value threshold of 10^− 10^.

GO and KEGG enrichment analysis was conducted with the help of ClusterProfiler v4.0.5 [72] to assess the enrichment of genes in specific GO terms or functional pathways (Fisher’s exact test, p-value < 0.05).

### Long noncoding RNA identification

After high-quality sequences were obtained, CPC v2 [73], CNCI v2 [74], PLEK v1.2 [75] and PfamScan v1.6 [76] were used with the default parameters to search for long noncoding RNAs (lncRNA) from the PacBio sequencing data. Transcripts predicted with coding potential by any of the four tools above were filtered out, and the remaining transcripts without coding potential and with a length of > 200 nt were identified as lncRNAs [13, 77].

### Validation of gene expression by RT-qPCR

cDNAs were synthesized by reverse transcription of total RNA from six xylem samples (DoSW1, DoSW2, DoSW3, DoTZ1, DoTZ2 and DoTZ3) of *D. odorifera*. Gene-specific primers for the targets (Supplementary Table 6) were designed with the help of Primer-BLAST [78]. Six genes were chosen to verify expression levels. RT-qPCR analysis was conducted with TB Green® Premix Ex Taq™ (Tli RNaseH Plus; Takara, Beijing, China) following the manufacturer’s recommendations. PCR amplification was performed in a preheated (95 °C) thermal cycler and incubated at 95 °C for 30 s, followed by 40 cycles of 95 °C for 5 s and 60 °C for 30 s. The actin gene served as an internal control for normalization [64]. The 2^–△△Ct^ method was used to calculate the expression levels of the genes against the internal control [79]. The nucleic acid sample isolated from the sapwood was set as the calibrator. Three technical replicates per sample were performed to ensure reproducibility and reliability.

## Conclusions

While the heartwood of *D. odorifera* has important application value, researches on the wood formation of *D. odorifera* hold great significance for cultivating better varieties. Post-transcriptional processes of genes’ expression play vital regulatory roles in plant development. This study reports the characterization of the global transcriptome of *D. odorifera*, including details on alternative polyadenylation (APA), alternative splicing (AS) and long noncoding RNAs (lncRNAs), in relation to xylem development. Here, the Illumina short reads and PacBio long reads were integrated to obtain full-length transcripts of *D. odorifera*, and AS and APA events along with lncRNAs were identified and characterized. We found that AS, APA and lncRNA could participate in the wood formation process of *D. odorifera*. We found that alternative splicing increases the transcriptome complexity of *D. odorifera*. Noticeably, evm.TU.scaffold_263.15, encoding isoliquiritigenin 2’-O-methyltransferase, experienced differential alternative last exon (AL) and might contribute to the accumulation of key heartwood substances of *D. odorifera*. DAS genes related to regulatory transcription factors were significantly enriched during GO analyses. These genes are likely to play important roles in regulation of xylem formation. Also, in the analysis of alternative polyadenylation, we found that APA genes were associated with wood formation. Specifically, we determined APA genes to be associated with terpenoid and flavonoid synthesis, such as *CHS*, *CHS-1B* and *HMGR*. Besides, some DE-APA genes were significantly enriched in the biological process involving cell wall macromolecule biosynthesis, metabolism, and biosynthesis of cell wall pectin. Furthermore, we found lncRNAs may play roles in xylem development by modulating the expression of protein-coding genes, such as *CHS1*, *RGTB2* and *MTERF4*. Overall, this study updates the gene annotation information in the genome of *D. odorifera*, provides insights into gene regulatory mechanisms in this species, and helps us in comprehending the underlying molecular regulatory mechanisms involved in wood formation of *D. odorifera*. Our study not only presents a useful genetic resource, it shall also benefit the future molecular-assisted selection in *D. odorifera*.

### Electronic supplementary material

Below is the link to the electronic supplementary material.

Supplementary Material 1

## Data Availability

The sequencing data in this paper have been deposited at the Genome Sequence Archive [80] in BIG Data Center [81], China National Center for Bioinformation / Beijing Institute of Genomics, Chinese Academy of Sciences (GSA: CRA012547, CRA006116) that are publicly accessible at https://ngdc.cncb.ac.cn/gsa.
