# Supplementary material for: Genome-wide characterization of post-transcriptional processes related to wood formation in Dalbergia odorifera
Source: BMC Genomics. 2024 Apr 16;25:372. doi: 10.1186/s12864-024-10300-7 (PMC11022335; doi:10.1186/s12864-024-10300-7)
Supplement: Supplementary file 1 — Supplementary Material 1 [file 12864_2024_10300_MOESM1_ESM.pdf]

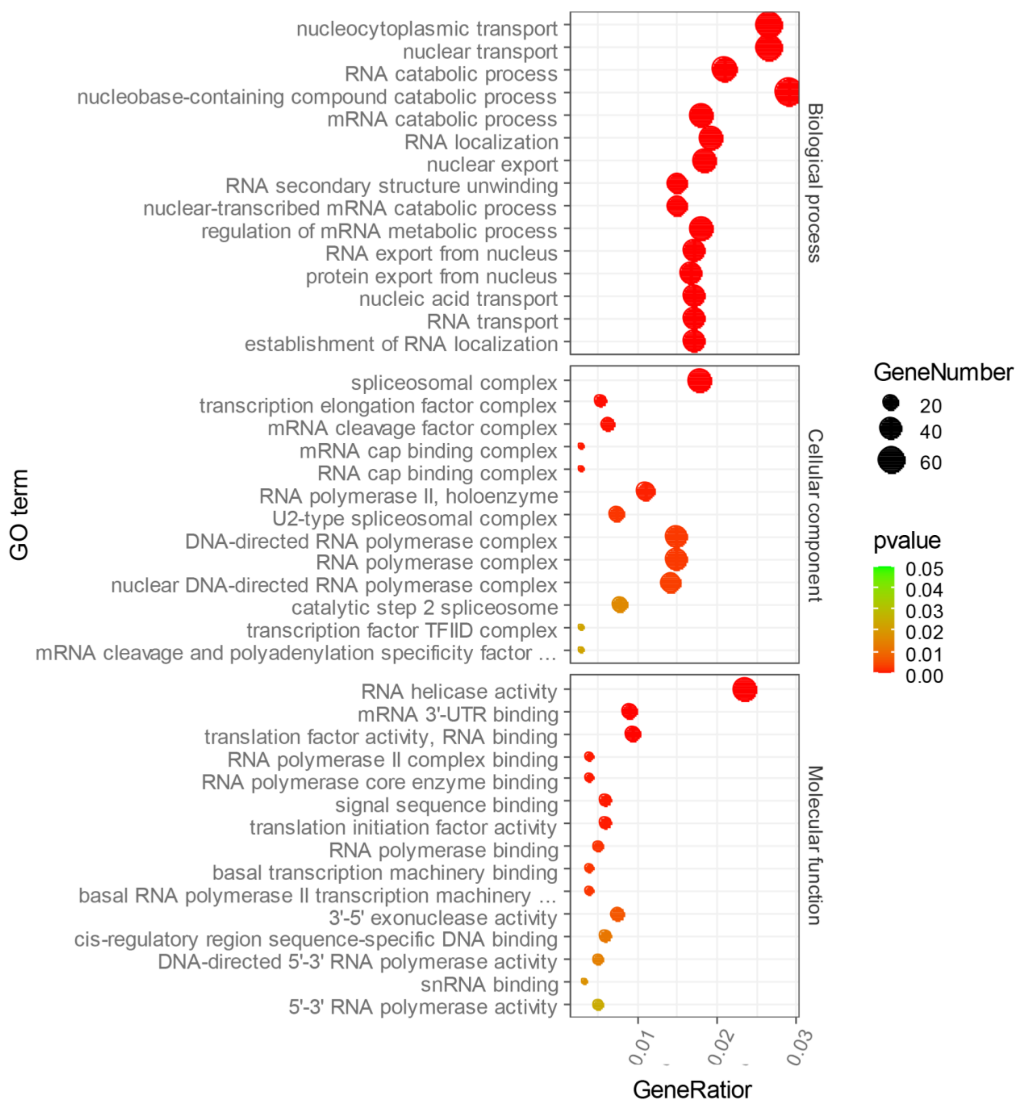

Supplementary Figure 1. APA genes involved in the regulation of gene transcription and post transcriptional regulation. Top 15 genes from each of the GO category are shown.

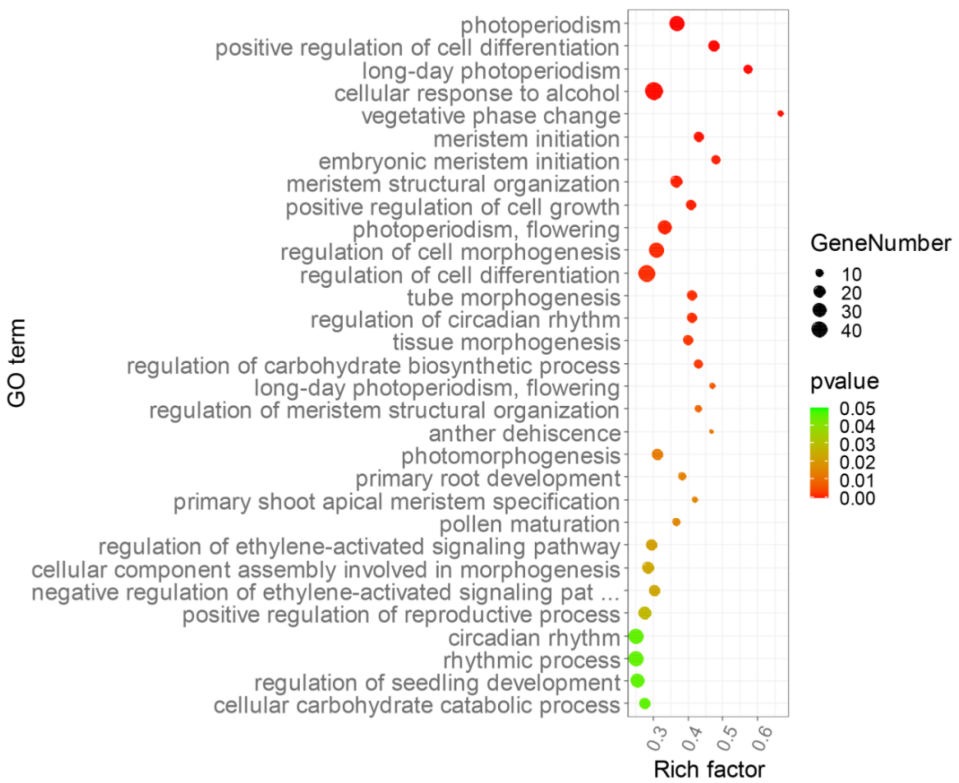

Supplementary Figure 2. APA genes enriched for the functions related to tissue differentiation and seedling development.

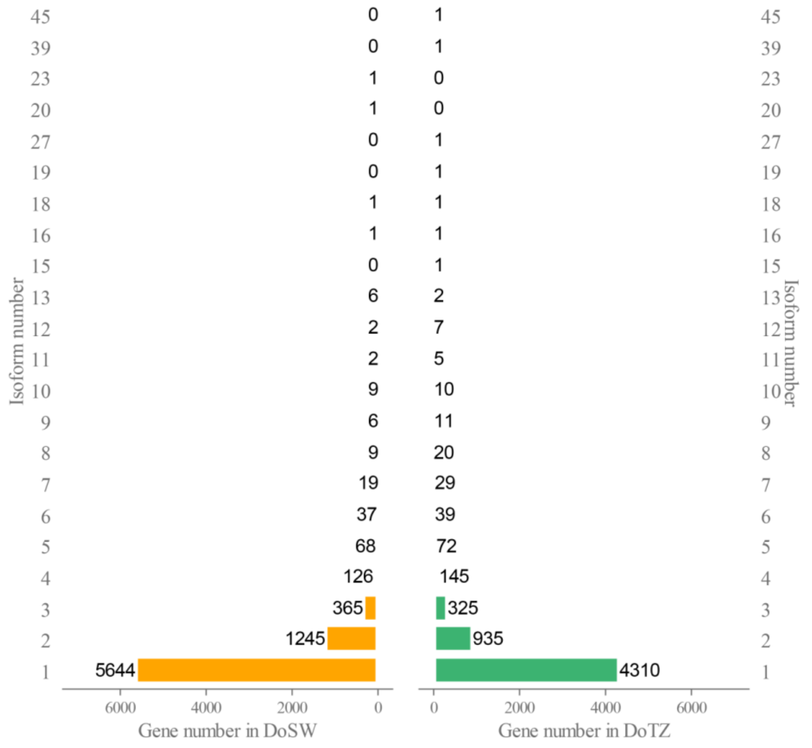

Supplementary Figure 3. Isoform number of genes identified by Iso-seq in the sapwood (DoSW) and the transition zone (DoTZ).

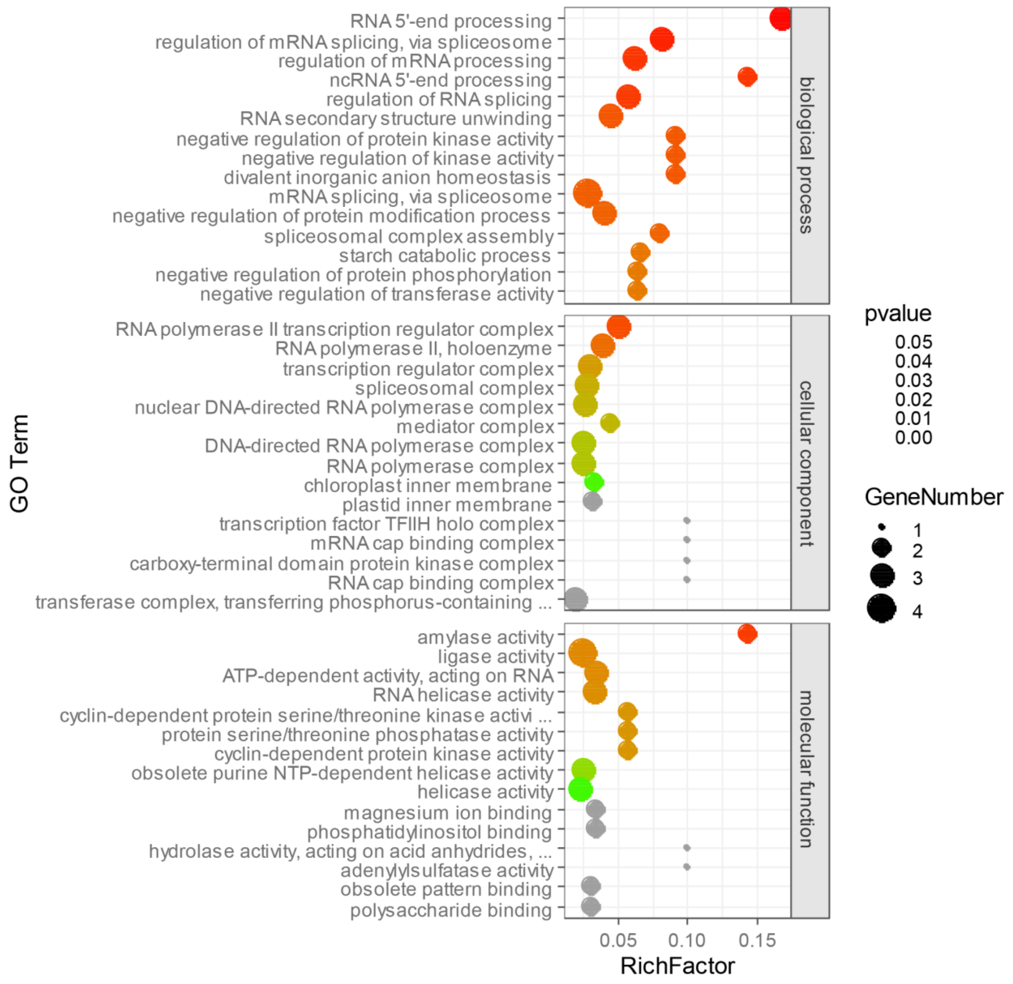

Supplementary Figure 4. Most significantly enriched GO (gene ontology) terms for DAS genes identified. Top 15 terms for each category are shown.

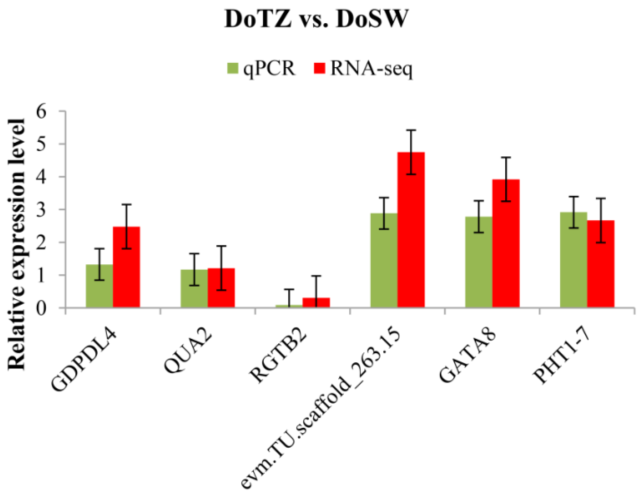

Supplementary Figure 5. The relative expression levels of six genes as determined by RT-qPCR and RNA-seq. Actin was used as an internal control for normalization in the RT-qPCR experiments.

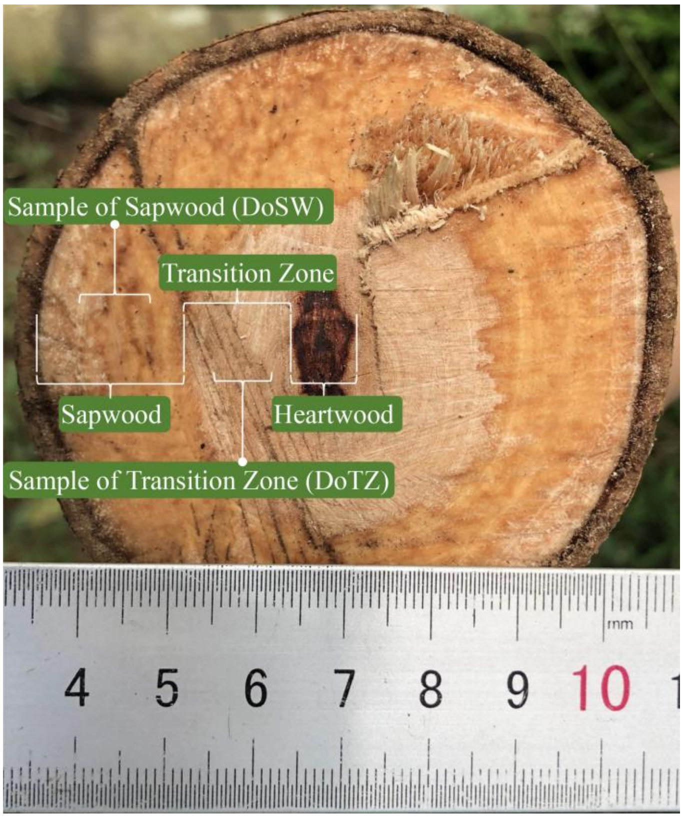

Supplementary Figure 6. A photograph of a cross-section of the tree with heartwood for sampling.

Supplementary Table 1. Differentially expressed APA genes that are related to terpenoid/flavonoid metabolism.

Gene_id	Gene Name	DoSW_group_fpkm	DoTZ_group_fpkm	DoTZ_readcount (DoTZvsDoSW)	DoSW_readcount (DoTZvsDoSW)	log2FoldChange (DoTZvsDoSW)	pval(DoTZvsDoSW)
evm.TU.scaffold_233.200	CHS	0.0049364	22.400312	1224.2507	0.2865186	12.061	$3.23x10^{-2}$
evm.TU.scaffold_48.236	CHS-1B	0.0248264	29.118294	1660.9482	1.8884276	9.7806	$4.87x10^{-2}$
evm.TU.scaffold_142.392	CCD8B	551.07813	0.9434377	53.308226	37190.094	-9.4463	$3.20x10^{-10}$
evm.TU.scaffold_416.4	CCD7	12.540183	0	0	819.83051	-26.28882	$7.79x10^{-7}$
evm.TU.scaffold_25.114	SDI1	12.460557	48.065555	2292.2551	732.86247	1.6452	$3.55x10^{-2}$
evm.TU.scaffold_40.1421	ABCG36	3.548057	12.385785	1519.7779	526.37041	1.5297	$4.76x10^{-2}$
evm.TU.scaffold_222.1492	--	9.9545623	0	0	804.34384	-26.26131	$1.30x10^{-5}$
evm.TU.scaffold_35.91	HMGR	0	62.59874	5635.6383	0	29.070004	$1.06x10^{-2}$
evm.TU.scaffold_36.899	CYP707A2	125.57646	3.5914265	215.38355	9441.5378	-5.454	$3.37x10^{-6}$

Supplementary Table 2. DE-APA genes related to cell wall synthesis, terpenoid biosynthetic and transcriptional regulation.

Gene_id	Gene Name	DoSW_ group_ fpkm	DoTZ_ group_ fpkm	DoTZ_readcount (DoTZvsDoSW)	DoSW_readcount (DoTZvsDoSW)	log2FoldChange (DoTZvsDoSW)	pval(DoTZ vsDoSW)	perc_diff	r	p.adjust
evm.TU.scaffold_111.270	GDPDL4	5.129	28.635	2740.975	706.730	1.956	0.426	0.275	-0.138	3.30×10^{-4}
evm.TU.scaffold_185.163	XEG113	26.604	38.449	3019.262	2506.198	0.269	0.731	0.222	0.161	2.10×10^{-5}
evm.TU.scaffold_206.364	GALT2	15.349	19.862	440.975	414.557	0.089	0.935	0.219	-0.291	2.95×10^{-3}
evm.TU.scaffold_40.1368	At1g72960	3.100	5.015	482.786	363.331	0.410	0.678	0.369	-0.423	1.57×10^{-2}
evm.TU.scaffold_413.180	QUA2	5.034	11.685	1180.514	724.860	0.704	0.599	0.245	0.247	2.39×10^{-3}
evm.TU.scaffold_5.1556	CSLG2	2.795	3.023	160.051	199.350	-0.317	0.886	0.333	0.457	8.15×10^{-3}
evm.TU.scaffold_5.1819	ARAD1	10.357	11.815	754.140	783.485	-0.055	0.862	0.200	0.334	7.38×10^{-3}
evm.TU.scaffold_100.697	DREB1F	16.723	90.843	3942.170	1028.505	1.938	0.431	0.237	-0.213	9.79×10^{-10}
evm.TU.scaffold_233.29	PNP1	4.264	8.172	960.530	586.174	0.713	0.415	0.381	-0.366	8.28×10^{-4}
evm.TU.scaffold_28.648	EDR1	1.654	1.790	160.997	186.195	-0.210	0.809	0.385	-0.418	3.00×10^{-2}
evm.TU.scaffold_369.38	RMS3	4.214	33.569	842.340	149.171	2.497	0.144	0.218	0.317	1.19×10^{-2}
evm.TU.scaffold_39.600	LUT2	2.604	3.647	251.459	227.668	0.143	0.835	0.652	-0.657	7.86×10^{-4}
evm.TU.scaffold_5.896	NCED5	3.601	2.461	67.268	105.935	-0.655	0.591	0.325	-0.491	2.77×10^{-5}
evm.TU.scaffold_248.80_ evm.TU.scaffold_248.81	NFXL2	13.842	16.756	1513.392	1449.212	0.063	0.921	0.261	-0.280	3.52×10^{-3}
evm.TU.scaffold_263.69	GATA8	3.166	47.922	2687.428	215.366	3.641	0.207	0.441	-0.252	4.21×10^{-5}
evm.TU.scaffold_28.104	BHLH66	9.957	17.879	1372.683	1077.835	0.349	0.745	0.273	-0.313	2.74×10^{-4}
evm.TU.scaffold_36.1161	SHL	62.302	78.334	3756.881	3726.675	0.012	0.963	0.200	-0.159	9.90×10^{-7}
evm.TU.scaffold_54.62	TCP8	16.513	17.303	1307.064	1465.184	-0.165	0.664	0.249	0.174	4.52×10^{-2}

Supplementary Table 3. Genes differentially expressed and also subjected to DAS.

Gene_id	Gene Name	Event_id	dosw-dotz_dPSI	dosw-dotz_p-val	DoSW_group_fpkm	DoTZ_group_fpkm	DoTZ_readcount(DoTZvsDoSW)	DoSW_readcount (DoTZvsDoSW)	log2FoldChange (DoTZvsDoSW)	pval(DoTZvsDoSW)
evm.TU.scaffold_461.545_evm.TU.scaffold_461.546	FRO8	SE:chr05:40673111-40672642:40673111-40673192:-	-0.397	0.010	23.140	2.824	226.557	2237.900	-3.304	7.74x10-6
evm.TU.scaffold_461.545_evm.TU.scaffold_461.546	FRO8	SE:chr05:40675670-40675819:40676001-40675819:-	-0.397	0.010	23.140	2.824	226.557	2237.900	-3.304	7.74x10-6
evm.TU.scaffold_461.545_evm.TU.scaffold_461.546	FRO8	RI:chr05:40674159:40674417-40674517:40674844:-	0.210	0.049	23.140	2.824	226.557	2237.900	-3.304	7.74x10-6
evm.TU.scaffold_46.117	PHT1-7	A5:chr06:18193160-18194794:18193005-18194794:+	-0.169	0.035	5.317	33.830	2449.694	418.235	2.550	9.31x10-3
Novelgene0421	--	AL:chr06:13782476-13782658:13783392:13782476-13805763:13806469:+	-0.609	0.001	84.877	19.613	681.011	3857.540	-2.502	1.07x10-2
evm.TU.scaffold_97.480	LAX1	A5:chr08:8477117-8477286:8477117-8477663:-	-0.165	0.022	30.077	9.553	838.626	3080.771	-1.877	3.17x10-2

Supplementary Table 4. Differentially expressed lncRNAs and their *cis*-regulated target genes.

LncRNA	mRNA	correlation	p.value
evm.TU.scaffold_427.16_novel02	evm.model.scaffold_427.16	0.979	6.41x10-4
evm.TU.scaffold_427.16_novel02	evm.model.scaffold_427.20	0.816	4.77x10-2
evm.TU.scaffold_427.16_novel02	evm.TU.scaffold_427.16_novel01	0.991	1.18x10-4
Novelgene0275_novel01	evm.model.scaffold_5.705	0.996	2.08x10-5
Novelgene0275_novel01	evm.model.scaffold_5.712	0.850	3.21x10-2
Novelgene0275_novel01	evm.model.scaffold_5.722	0.998	5.73x10-6
Novelgene0275_novel01	evm.TU.scaffold_5.709_novel01	0.876	2.19x10-2
Novelgene0275_novel01	evm.TU.scaffold_5.710_novel01	-0.886	1.87x10-2
Novelgene1027_novel01	evm.model.scaffold_396.327	0.946	4.31x10-3
Novelgene1027_novel01	evm.model.scaffold_396.329	-0.843	3.52x10-2
Novelgene1027_novel01	evm.model.scaffold_396.330	-0.900	1.44x10-2
Novelgene1027_novel01	evm.model.scaffold_396.331	-0.965	1.84x10-3
Novelgene1027_novel01	evm.model.scaffold_396.335	0.842	3.56x10-2
Novelgene1027_novel01	evm.model.scaffold_396.341	0.826	4.27x10-2
Novelgene1027_novel01	evm.model.scaffold_396.345	0.835	3.87x10-2
Novelgene1027_novel01	evm.model.scaffold_396.346	0.946	4.31x10-3

Supplementary Table 5. *Trans*-regulated targets of differentially expressed lncRNAs.

LncRNA	mRNA	Length_LncRNA	Length_mRNA	dG	ndG	correlation	p.value
evm.TU.scaffold_375.540_novel01	evm.model.scaffold_159.18	1307	369	-36.89	-0.1011	0.814	$4.89x10^{-2}$
evm.TU.scaffold_375.540_novel01	evm.model.scaffold_18.321	1307	219	-30.85	-0.2043	0.981	$5.66x10^{-4}$
evm.TU.scaffold_375.540_novel01	evm.model.scaffold_185.150	1307	237	-29.17	-0.1527	0.981	$5.66x10^{-4}$
evm.TU.scaffold_375.540_novel01	evm.model.scaffold_205.39	1307	249	-25.61	-0.1033	0.903	$1.36x10^{-2}$
evm.TU.scaffold_375.540_novel01	evm.model.scaffold_222.252	1307	396	-52.49	-0.1446	0.868	$2.49x10^{-2}$
evm.TU.scaffold_375.540_novel01	evm.model.scaffold_241.13	1307	270	-32.26	-0.128	0.981	$5.66x10^{-4}$
evm.TU.scaffold_375.540_novel01	evm.model.scaffold_25.153	1307	346	-34.46	-0.1104	0.930	$7.14x10^{-3}$
evm.TU.scaffold_375.540_novel01	evm.model.scaffold_263.203	1307	288	-29.94	-0.1085	0.856	$2.96x10^{-2}$
evm.TU.scaffold_375.540_novel01	evm.model.scaffold_264.11	1307	282	-37.89	-0.151	0.981	$5.66x10^{-4}$
evm.TU.scaffold_375.540_novel01	evm.model.scaffold_28.856	1307	378	-36.82	-0.1049	0.887	$1.85x10^{-2}$
evm.TU.scaffold_375.540_novel01	evm.model.scaffold_28.988	1307	399	-21.82	-0.1006	0.909	$1.21x10^{-2}$
evm.TU.scaffold_375.540_novel01	evm.model.scaffold_29.648	1307	396	-18.68	-0.1146	0.981	$5.66x10^{-4}$
evm.TU.scaffold_375.540_novel01	evm.model.scaffold_299.155	1307	349	-33.59	-0.1021	0.981	$5.66x10^{-4}$
evm.TU.scaffold_375.540_novel01	evm.model.scaffold_320.106	1307	291	-26.94	-0.1028	0.997	$1.79x10^{-5}$
evm.TU.scaffold_375.540_novel01	evm.model.scaffold_378.76	1307	291	-34.77	-0.1302	0.966	$1.67x10^{-3}$
evm.TU.scaffold_375.540_novel01	evm.model.scaffold_40.1498	1307	228	-14.62	-0.1142	0.981	$5.66x10^{-4}$
evm.TU.scaffold_375.540_novel01	evm.model.scaffold_40.1644	1307	396	-39.81	-0.1073	0.981	$5.66x10^{-4}$
evm.TU.scaffold_375.540_novel01	evm.model.scaffold_41.13	1307	357	-23.87	-0.1148	0.981	$5.66x10^{-4}$
evm.TU.scaffold_375.540_novel01	evm.model.scaffold_416.497	1307	309	-37.65	-0.1354	0.929	$7.42x10^{-3}$
evm.TU.scaffold_375.540_novel01	evm.model.scaffold_42.315	1307	336	-30.39	-0.1044	0.820	$4.56x10^{-2}$
evm.TU.scaffold_375.540_novel01	evm.model.scaffold_457.123	1307	342	-32.9	-0.1035	0.981	$5.66x10^{-4}$
evm.TU.scaffold_375.540_novel01	evm.model.scaffold_46.621	1307	231	-34.67	-0.1691	0.981	$5.66x10^{-4}$
evm.TU.scaffold_375.540_novel01	evm.model.scaffold_461.339	1307	255	-28.33	-0.118	0.930	$7.14x10^{-3}$
evm.TU.scaffold_375.540_novel01	evm.model.scaffold_5.1164	1307	330	-20.54	-0.1698	0.981	$5.66x10^{-4}$

evm.TU.scaffold_375.540_novel01	evm.model.scaffold_6.4	1307	315	-28.37	-0.1002	0.891	$1.72x10^{-2}$
evm.TU.scaffold_375.540_novel01	evm.model.scaffold_7.134	1307	309	-27.9	-0.1167	0.970	$1.37x10^{-3}$
evm.TU.scaffold_375.540_novel01	evm.model.scaffold_9.33	1307	324	-31.79	-0.1029	0.981	$5.66x10^{-4}$
evm.TU.scaffold_375.540_novel01	evm.model.scaffold_97.455	1307	189	-16.67	-0.1174	0.981	$5.66x10^{-4}$
evm.TU.scaffold_375.540_novel01	evm.model.scaffold_97.597	1307	297	-39.86	-0.1429	0.928	$7.69x10^{-3}$
evm.TU.scaffold_427.16_novel02	evm.model.scaffold_100.786	217	4208	-16.09	-0.1052	0.899	$1.49x10^{-2}$
evm.TU.scaffold_427.16_novel02	evm.model.scaffold_100.871	217	1653	-14.37	-0.115	-0.845	$3.42x10^{-2}$
evm.TU.scaffold_427.16_novel02	evm.model.scaffold_14.160	217	2244	-15.6	-0.1006	0.970	$1.33x10^{-3}$
evm.TU.scaffold_427.16_novel02	evm.model.scaffold_14.222	217	4909	-14.08	-0.11	0.931	$7.03x10^{-3}$
evm.TU.scaffold_427.16_novel02	evm.model.scaffold_14.40	217	2020	-13.45	-0.1093	0.892	$1.68x10^{-2}$
evm.TU.scaffold_427.16_novel02	evm.model.scaffold_140.5	217	264	-11.19	-0.1142	-0.829	$4.12x10^{-2}$
evm.TU.scaffold_427.16_novel02	evm.model.scaffold_176.148	217	4757	-12.84	-0.2568	0.849	$3.23x10^{-2}$
evm.TU.scaffold_427.16_novel02	evm.model.scaffold_222.643	217	1882	-13.34	-0.1131	-0.863	$2.67x10^{-2}$
evm.TU.scaffold_427.16_novel02	evm.model.scaffold_233.22	217	432	-8.17	-0.1021	0.824	$4.39x10^{-2}$
evm.TU.scaffold_427.16_novel02	evm.model.scaffold_248.366	217	1075	-12.11	-0.1682	0.952	$3.46x10^{-3}$
evm.TU.scaffold_427.16_novel02	evm.model.scaffold_274.19	217	357	-6.22	-0.2006	-0.850	$3.21x10^{-2}$
evm.TU.scaffold_427.16_novel02	evm.model.scaffold_299.239	217	578	-9.59	-0.1314	-0.876	$2.21x10^{-2}$
evm.TU.scaffold_427.16_novel02	evm.model.scaffold_307.161	217	1138	-12.58	-0.1233	-0.847	$3.34x10^{-2}$
evm.TU.scaffold_427.16_novel02	evm.model.scaffold_35.677	217	288	-10.14	-0.137	-0.975	$9.44x10^{-4}$
evm.TU.scaffold_427.16_novel02	evm.model.scaffold_350.108	217	2299	-16.52	-0.1139	-0.929	$7.38x10^{-3}$
evm.TU.scaffold_427.16_novel02	evm.model.scaffold_36.1235	217	1433	-17.55	-0.1009	0.825	$4.35x10^{-2}$
evm.TU.scaffold_427.16_novel02	evm.model.scaffold_375.360	217	2046	-17.4	-0.1192	0.890	$1.74x10^{-2}$
evm.TU.scaffold_427.16_novel02	evm.model.scaffold_375.97	217	4454	-17.37	-0.1059	0.836	$3.82x10^{-2}$
evm.TU.scaffold_427.16_novel02	evm.model.scaffold_376.182	217	507	-10.88	-0.136	-0.829	$4.14x10^{-2}$
evm.TU.scaffold_427.16_novel02	evm.model.scaffold_376.281	217	492	-11.5	-0.1337	0.824	$4.37x10^{-2}$
evm.TU.scaffold_427.16_novel02	evm.model.scaffold_40.357	217	951	-18.9	-0.1005	0.904	$1.35x10^{-2}$

evm.TU.scaffold_427.16_novel02	evm.model.scaffold_416.280	217	306	-7.1	-0.1392	0.890	$1.74x10^{-2}$
evm.TU.scaffold_427.16_novel02	evm.model.scaffold_416.32	217	1281	-10.3	-0.1451	0.927	$7.79x10^{-3}$
evm.TU.scaffold_427.16_novel02	evm.model.scaffold_44.226	217	525	-10.63	-0.1456	0.848	$3.31x10^{-2}$
evm.TU.scaffold_427.16_novel02	evm.model.scaffold_457.456	217	2199	-15.2	-0.1034	0.882	$2.00x10^{-2}$
evm.TU.scaffold_427.16_novel02	evm.model.scaffold_457.504	217	630	-10.84	-0.1095	0.978	$7.32x10^{-4}$
evm.TU.scaffold_427.16_novel02	evm.model.scaffold_461.898	217	1278	-15.93	-0.1008	0.845	$3.41x10^{-2}$
evm.TU.scaffold_427.16_novel02	evm.model.scaffold_5.1186	217	1899	-14.62	-0.1282	0.949	$3.78x10^{-3}$
evm.TU.scaffold_427.16_novel02	evm.model.scaffold_5.359	217	1244	-15.53	-0.1028	0.865	$2.62x10^{-2}$
evm.TU.scaffold_427.16_novel02	evm.model.scaffold_5.865	217	2605	-14.51	-0.1423	0.863	$2.70x10^{-2}$
evm.TU.scaffold_427.16_novel02	evm.model.scaffold_5.954	217	2648	-11.72	-0.1149	-0.863	$2.68x10^{-2}$
evm.TU.scaffold_427.16_novel02	evm.model.scaffold_51.15	217	663	-9.4	-0.1306	0.907	$1.27x10^{-2}$
evm.TU.scaffold_427.16_novel02	evm.model.scaffold_69.430.2	217	1602	-16.42	-0.114	-0.912	$1.14x10^{-2}$
evm.TU.scaffold_427.16_novel02	evm.model.scaffold_97.510	217	1047	-11.11	-0.2096	0.840	$3.61x10^{-2}$
evm.TU.scaffold_427.16_novel02	evm.TU.scaffold_100.659_novel01	217	3597	-17.81	-0.1018	0.839	$3.69x10^{-2}$
evm.TU.scaffold_427.16_novel02	evm.TU.scaffold_100.758_novel01	217	2946	-12.77	-0.168	0.934	$6.45x10^{-3}$
evm.TU.scaffold_427.16_novel02	evm.TU.scaffold_111.334_novel01	217	1034	-15.96	-0.105	0.875	$2.25x10^{-2}$
evm.TU.scaffold_427.16_novel02	evm.TU.scaffold_206.593_novel01	217	1199	-13.15	-0.1044	0.914	$1.08x10^{-2}$
evm.TU.scaffold_427.16_novel02	evm.TU.scaffold_222.1326_novel01	217	2025	-11.48	-0.1053	0.861	$2.78x10^{-2}$
evm.TU.scaffold_427.16_novel02	evm.TU.scaffold_222.1892_novel01	217	3673	-12.77	-0.1388	0.930	$7.16x10^{-3}$
evm.TU.scaffold_427.16_novel02	evm.TU.scaffold_264.98_novel01	217	4535	-12.74	-0.1699	0.832	$3.97x10^{-2}$
evm.TU.scaffold_427.16_novel02	evm.TU.scaffold_40.301_novel06	217	3306	-14.65	-0.1181	0.922	$8.93x10^{-3}$
evm.TU.scaffold_427.16_novel02	evm.TU.scaffold_44.120_novel01	217	3810	-24.16	-0.122	0.820	$4.58x10^{-2}$
evm.TU.scaffold_427.16_novel02	evm.TU.scaffold_5.81_novel04	217	2763	-15.55	-0.1119	-0.868	$2.49x10^{-2}$
evm.TU.scaffold_427.16_novel02	evm.TU.scaffold_54.313_novel01	217	2987	-10.93	-0.1539	0.877	$2.17x10^{-2}$
evm.TU.scaffold_427.16_novel02	evm.TU.scaffold_56.56_novel02	217	2220	-9.83	-0.1143	-0.927	$7.77x10^{-3}$
evm.TU.scaffold_427.16_novel02	evm.TU.scaffold_62.20_novel01	217	1411	-20.11	-0.1006	-0.861	$2.77x10^{-2}$

evm.TU.scaffold_427.16_novel02	evm.TU.scaffold_62.52_novel10	217	1616	-11.93	-0.1065	-0.907	$1.26x10^{-2}$
Novelgene0275_novel01	evm.model.scaffold_100.1061	719	1785	-22.09	-0.1067	0.847	$3.33x10^{-2}$
Novelgene0275_novel01	evm.model.scaffold_100.806	719	771	-17.56	-0.2509	0.966	$1.73x10^{-3}$
Novelgene0275_novel01	evm.model.scaffold_142.107	719	772	-19.15	-0.1154	0.915	$1.04x10^{-2}$
Novelgene0275_novel01	evm.model.scaffold_159.24	719	216	-20.92	-0.1189	0.981	$5.49x10^{-4}$
Novelgene0275_novel01	evm.model.scaffold_273.371	719	591	-32.71	-0.1032	0.998	$5.73x10^{-6}$
Novelgene0275_novel01	evm.model.scaffold_307.275	719	1269	-30.57	-0.1176	0.981	$5.45x10^{-4}$
Novelgene0275_novel01	evm.model.scaffold_394.136.1	719	1529	-16.51	-0.1353	0.904	$1.35x10^{-2}$
Novelgene0275_novel01	evm.model.scaffold_394.5	719	771	-25.47	-0.1044	0.857	$2.91x10^{-2}$
Novelgene0275_novel01	evm.model.scaffold_396.257	719	201	-14.88	-0.1127	0.905	$1.32x10^{-2}$
Novelgene0275_novel01	evm.model.scaffold_40.483	719	394	-20.48	-0.1004	0.918	$9.78x10^{-3}$
Novelgene0275_novel01	evm.model.scaffold_42.304	719	177	-15.7	-0.1539	0.998	$5.73x10^{-6}$
Novelgene0275_novel01	evm.model.scaffold_44.107	719	910	-17.35	-0.1535	0.890	$1.75x10^{-2}$
Novelgene0275_novel01	evm.model.scaffold_5.163	719	243	-23.64	-0.1032	0.998	$5.73x10^{-6}$
Novelgene0275_novel01	evm.TU.scaffold_100.1654_novel01	719	1290	-18.15	-0.1476	0.939	$5.41x10^{-3}$
Novelgene0275_novel01	evm.TU.scaffold_320.577_novel01	719	4756	-240.51	-0.365	0.932	$6.76x10^{-3}$
Novelgene0275_novel01	evm.TU.scaffold_394.360_novel01	719	2046	-18.87	-0.1158	0.945	$4.49x10^{-3}$
Novelgene0275_novel01	evm.TU.scaffold_40.1308_novel01	719	1997	-305.45	-0.4427	0.880	$2.06x10^{-2}$
Novelgene0275_novel01	Novelgene0108_novel01	719	5331	-343.01	-0.4838	0.999	$1.06x10^{-6}$
Novelgene0275_novel01	Novelgene0795_novel01	719	796	-22.36	-0.1016	0.964	$1.93x10^{-3}$
Novelgene0310_novel01	evm.model.scaffold_227.34	2605	207	-16.9	-0.103	0.910	$1.17x10^{-2}$
Novelgene0310_novel01	evm.model.scaffold_263.59	2605	384	-26.11	-0.1116	0.910	$1.17x10^{-2}$
Novelgene0310_novel01	evm.model.scaffold_274.7	2605	333	-13.89	-0.1177	0.910	$1.17x10^{-2}$
Novelgene0310_novel01	evm.model.scaffold_28.46	2605	258	-19.54	-0.1206	0.910	$1.17x10^{-2}$
Novelgene0310_novel01	evm.model.scaffold_35.1192	2605	516	-21.77	-0.1105	0.892	$1.69x10^{-2}$
Novelgene0310_novel01	evm.model.scaffold_394.533	2605	904	-21.47	-0.1256	-0.824	$4.37x10^{-2}$

Novelgene0310_novel01	evm.model.scaffold_416.650	2605	1212	-14.02	-0.1078	-0.821	$4.54x10^{-2}$
Novelgene0310_novel01	evm.model.scaffold_48.53	2605	204	-15.35	-0.1312	0.964	$1.93x10^{-3}$
Novelgene0310_novel01	evm.TU.scaffold_100.1452_novel01	2605	2215	-21.2	-0.2038	-0.832	$4.02x10^{-2}$
Novelgene0310_novel01	evm.TU.scaffold_222.361_novel01	2605	2611	-18.06	-0.3344	0.893	$1.66x10^{-2}$
Novelgene0310_novel01	evm.TU.scaffold_222.491_novel01	2605	3810	-23.3	-0.1752	0.894	$1.63x10^{-2}$
Novelgene0310_novel01	evm.TU.scaffold_375.116_novel01	2605	878	-20.35	-0.1197	-0.917	$1.01x10^{-2}$
Novelgene0310_novel01	evm.TU.scaffold_375.566_novel01	2605	2460	-16.69	-0.1098	0.892	$1.68x10^{-2}$
Novelgene0310_novel01	evm.TU.scaffold_396.362_novel01	2605	3227	-14.99	-0.153	0.910	$1.17x10^{-2}$
Novelgene0310_novel01	Novelgene0156_novel01	2605	913	-23.35	-0.1249	0.980	$6.06x10^{-4}$
Novelgene0310_novel01	Novelgene0573_novel01	2605	5074	-12.28	-0.2856	0.945	$4.40x10^{-3}$
Novelgene0310_novel01	Novelgene0660_novel01	2605	1131	-22	-0.1294	0.873	$2.31x10^{-2}$
Novelgene0867_novel01	evm.model.scaffold_100.1886	4302	420	-47.79	-0.1274	0.843	$3.51x10^{-2}$
Novelgene0867_novel01	evm.model.scaffold_100.454	4302	180	-16.95	-0.1169	0.987	$2.37x10^{-4}$
Novelgene0867_novel01	evm.model.scaffold_159.73	4302	306	-33.35	-0.1119	0.942	$4.97x10^{-3}$
Novelgene0867_novel01	evm.model.scaffold_222.1370	4302	228	-26.71	-0.1309	0.999	$7.88x10^{-7}$
Novelgene0867_novel01	evm.model.scaffold_222.597	4302	360	-42.67	-0.1451	0.907	$1.26x10^{-2}$
Novelgene0867_novel01	evm.model.scaffold_227.34	4302	207	-19.97	-0.1079	0.987	$2.37x10^{-4}$
Novelgene0867_novel01	evm.model.scaffold_241.265	4302	243	-29.51	-0.124	0.817	$4.71x10^{-2}$
Novelgene0867_novel01	evm.model.scaffold_25.35	4302	357	-39.51	-0.1216	0.987	$2.37x10^{-4}$
Novelgene0867_novel01	evm.model.scaffold_263.204	4302	294	-42.81	-0.1529	0.922	$8.81x10^{-3}$
Novelgene0867_novel01	evm.model.scaffold_28.46	4302	258	-29.55	-0.1285	0.987	$2.37x10^{-4}$
Novelgene0867_novel01	evm.model.scaffold_290.507	4302	231	-24.15	-0.1123	0.987	$2.37x10^{-4}$
Novelgene0867_novel01	evm.model.scaffold_311.29	4302	423	-43.01	-0.1225	0.866	$2.57x10^{-2}$
Novelgene0867_novel01	evm.model.scaffold_324.41	4302	279	-29.71	-0.1084	0.926	$7.92x10^{-3}$
Novelgene0867_novel01	evm.model.scaffold_34.107	4302	315	-37.25	-0.125	0.872	$2.36x10^{-2}$
Novelgene0867_novel01	evm.model.scaffold_34.40	4302	447	-50.04	-0.1132	0.888	$1.82x10^{-2}$

Novelgene0867_novel01	evm.model.scaffold_340.70	4302	225	-28.8	-0.1358	0.916	$1.03x10^{-2}$
Novelgene0867_novel01	evm.model.scaffold_36.1249	4302	282	-40.43	-0.1789	0.973	$1.11x10^{-3}$
Novelgene0867_novel01	evm.model.scaffold_36.559	4302	282	-32.77	-0.1183	0.853	$3.09x10^{-2}$
Novelgene0867_novel01	evm.model.scaffold_37.99	4302	258	-37.09	-0.1627	0.987	$2.37x10^{-4}$
Novelgene0867_novel01	evm.model.scaffold_375.79	4302	204	-31.25	-0.1653	0.890	$1.73x10^{-2}$
Novelgene0867_novel01	evm.model.scaffold_378.203	4302	354	-36.85	-0.1117	0.952	$3.35x10^{-3}$
Novelgene0867_novel01	evm.model.scaffold_386.143	4302	258	-27.44	-0.1148	0.836	$3.83x10^{-2}$
Novelgene0867_novel01	evm.model.scaffold_396.397	4302	381	-41.44	-0.1126	0.937	$5.90x10^{-3}$
Novelgene0867_novel01	evm.model.scaffold_4.39	4302	294	-42.04	-0.1512	0.828	$4.19x10^{-2}$
Novelgene0867_novel01	evm.model.scaffold_4.81	4302	378	-40.38	-0.1231	0.987	$2.37x10^{-4}$
Novelgene0867_novel01	evm.model.scaffold_40.80	4302	306	-47.18	-0.1562	0.987	$2.37x10^{-4}$
Novelgene0867_novel01	evm.model.scaffold_416.107	4302	267	-41.67	-0.1911	0.828	$4.17x10^{-2}$
Novelgene0867_novel01	evm.model.scaffold_44.159	4302	294	-40.59	-0.1549	0.830	$4.08x10^{-2}$
Novelgene0867_novel01	evm.model.scaffold_461.203	4302	324	-32.61	-0.1094	0.987	$2.37x10^{-4}$
Novelgene0867_novel01	evm.model.scaffold_461.417	4302	174	-17.49	-0.1136	0.907	$1.26x10^{-2}$
Novelgene0867_novel01	evm.model.scaffold_461.570	4302	321	-31.91	-0.1019	0.833	$3.96x10^{-2}$
Novelgene0867_novel01	evm.model.scaffold_48.53	4302	204	-34.13	-0.169	0.969	$1.44x10^{-3}$
Novelgene0867_novel01	evm.model.scaffold_5.1727	4302	324	-37.25	-0.1198	0.899	$1.46x10^{-2}$
Novelgene0867_novel01	evm.model.scaffold_5.672	4302	252	-31.27	-0.1454	-0.921	$9.06x10^{-3}$
Novelgene0867_novel01	evm.model.scaffold_5.784	4302	489	-46.82	-0.1027	0.932	$6.85x10^{-3}$
Novelgene1027_novel01	evm.model.scaffold_396.170	3080	1024	-24.61	-0.103	0.853	$3.07x10^{-2}$
Novelgene1027_novel01	evm.model.scaffold_396.257	3080	201	-17.99	-0.1011	0.865	$2.59x10^{-2}$
Novelgene1027_novel01	evm.model.scaffold_419.258	3080	156	-12.56	-0.1005	0.946	$4.31x10^{-3}$
Novelgene1027_novel01	evm.model.scaffold_42.468	3080	219	-15.53	-0.1022	0.869	$2.46x10^{-2}$
Novelgene1027_novel01	evm.model.scaffold_46.673	3080	219	-21.71	-0.1131	0.946	$4.31x10^{-3}$
Novelgene1027_novel01	evm.TU.scaffold_36.932_novel01	1839	1266	-25.54	-0.1476	0.876	$2.23x10^{-2}$

Novelgene1027_novel01	GeneWise.13768.1	3080	174	-15.69	-0.1414	0.946	4.31×10^{-3}

Supplementary Table 6. Primers used for RT-qPCR assays in this study.

Gene	Primer pairs
RGTB2	F:TCAGGGGATATGTGGGGTGA
	R:GTGACCCCGTTAGAGCAAGG
evm.TU.scaffold_263.15	F:TGCAGTGCTCCAAGTTGGTT
	R:TTAGGGAGGTCAGAGGGGTG
GATA8	F:GCTGCCTCACCAGAACTCAT
	R:TATTCCCCCAGTCCAACCCT
PHT1-7	F:TAAGGCAGGAGCCATTGTGG
	R:TCTGGCACCAACAAGGTGAA
GDPDL4	F:CCACCTTTGCCTGCTGTTTC
	R:GGGCGATGATGTCCCTCAAA
QUA2	F:GCCCTTTACCCCTGAGGTTC
	R:ATCCAGTGAGCGCAAGAACA
Actin	F:CAATGAATTGCGTGTTGCT
	R:ATACCAGTTGTGCGACCACTT
